# Pathway signatures derived from on-treatment tumor specimens predict response to anti-PD1 blockade in metastatic melanoma

**DOI:** 10.1038/s41467-021-26299-4

**Published:** 2021-10-15

**Authors:** Kuang Du, Shiyou Wei, Zhi Wei, Dennie T. Frederick, Benchun Miao, Tabea Moll, Tian Tian, Eric Sugarman, Dmitry I. Gabrilovich, Ryan J. Sullivan, Lunxu Liu, Keith T. Flaherty, Genevieve M. Boland, Meenhard Herlyn, Gao Zhang

**Affiliations:** 1 Department of Computer Science, Ying Wu College of Computing, New Jersey Institute of Technology, Newark, NJ 07102 USA; 2 Department of Thoracic Surgery, West China Hospital, Sichuan University, 610041 Chengdu, Sichuan China; 3 Department of Neurosurgery, Duke University School of Medicine, Durham, NC 27710 USA; 4 The Preston Robert Tisch Brain Tumor Center, Duke University Medical Center, Durham, NC 27710 USA; 5 Massachusetts General Hospital Cancer Center, Boston, MA 02114 USA; 6 Department of Surgery, Massachusetts General Hospital, Boston, MA 02114 USA; 7 Philadelphia College of Osteopathic Medicine, Philadelphia, PA 19131 USA; 8 Cancer Immunology, AstraZeneca, Gaithersburg, MD 20878 USA; 9 Molecular and Cellular Oncogenesis Program and Melanoma Research Center, The Wistar Institute, Philadelphia, PA 19104 USA; 10 Department of Pathology, Duke University School of Medicine, Durham, NC 27710 USA

**Keywords:** Cancer immunotherapy, Melanoma, Predictive markers

## Abstract

Both genomic and transcriptomic signatures have been developed to predict responses of metastatic melanoma to immune checkpoint blockade (ICB) therapies; however, most of these signatures are derived from pre-treatment biopsy samples. Here, we build pathway-based super signatures in pre-treatment (PASS-PRE) and on-treatment (PASS-ON) tumor specimens based on transcriptomic data and clinical information from a large dataset of metastatic melanoma treated with anti-PD1-based therapies as the training set. Both PASS-PRE and PASS-ON signatures are validated in three independent datasets of metastatic melanoma as the validation set, achieving area under the curve (AUC) values of 0.45–0.69 and 0.85–0.89, respectively. We also combine all test samples and obtain AUCs of 0.65 and 0.88 for PASS-PRE and PASS-ON signatures, respectively. When compared with existing signatures, the PASS-ON signature demonstrates more robust and superior predictive performance across all four datasets. Overall, we provide a framework for building pathway-based signatures that is highly and accurately predictive of response to anti-PD1 therapies based on on-treatment tumor specimens. This work would provide a rationale for applying pathway-based signatures derived from on-treatment tumor samples to predict patients’ therapeutic response to ICB therapies.

## Abstract (web summary)

Gene signatures that predict response to immune checkpoint blockade (ICB) therapies in melanoma have been based on preclinical models and pre-treatment samples. Here the authors develop pathway-based signatures to predict ICB response in melanoma using on-treatment samples, leading to improved performance.

## Introduction

While remarkable success of immune checkpoint blockade (ICB) therapies has been achieved in treating patients with metastatic melanoma and many other types of cancers, only a subset of patients have derived a long-term benefit and achieved a durable clinical response^1–4^. The lack of robust clinical tools to guide ICB therapies not only fails to triage patients but also leads to its overuse, which may incur considerable side effects and costs. Therefore, it is necessary to identify predictive biomarkers of response to ICB therapies in order to inform and optimize therapeutic decisions.

Previous genomic and transcriptomic studies have identified numerous biomarkers that predict response of metastatic melanoma to ICB therapies^5–20^. These predictive biomarkers include tumor mutational burden (TMB) and neoantigens load^5,6,9,10,21,22^, HLA-I genotype^23,24^, cytolytic activity^13^, aneuploidy^12^, and T-cell repertoire^11^. In addition, gene expression signatures like immune-predictive score (IMPRES) and IFN-γ-responsive genes expressed in tumors or tumor immune microenvironments (TiME) have also been implicated in predicting response of metastatic melanoma to ICB therapies^7,8,11–20^. IMPRES consisting of 15 immune genes was developed and validated in several independent datasets that showed a high predictive power of response to ICB therapies in metastatic melanoma. A pan-tumor T-cell-inflamed gene expression profile (GEP) consisting of 18 IFN-γ-responsive genes was validated and confirmed to predict response to ICB therapy in pretreatment tumor specimens from nine types of cancers, including melanoma^17^. MHC-I/II gene signatures can also be utilized as biomarkers to predict response to ICB therapy in melanoma^19,25,26^. Among those signatures, TMB and gene expression signatures have been widely used in many studies to predict response to ICB therapies. However, these studies are limited in most cases because these predictive signatures have often been constructed based on preclinical models, clinical cohorts with only pre-treatment biopsies, peripheral blood samples, and NanoString RNA panel with a limited number of transcripts as opposed to whole-transcriptomic RNA sequencing (RNAseq) data^8,27–29^. Indeed, most of the predictive signatures for ICB therapies failed to be validated across cohorts because of batch effect, lack of reproducibility or other reasons, which led to debates in this field^30–33^. For example, Carter et al. have raised their concerns about IMPRES^14^, stating that it did not reproducibly predict response to ICB therapies in metastatic melanoma^30^. Xiao et al. have also raised the reproducibility question of ImmuneCells.Sig^34^ across different datasets of RNAseq data^32^.

Indeed, it is conceptually and technically challenging to develop a reliable and robust signature to predict response to ICB therapies. The reproducibility of a signature across independent datasets and potentially various cancer types is an essential requirement before it can be widely used in clinical practice. Therefore, we have reasoned that single genes or predictive signatures constructed with only a limited number of genes may reduce the reproducibility and generalizability due to the noise inherent from gene expression data and batch effects^35^. Given this reason, some studies have developed predictive signatures based on pathways to mitigate batch effects and other technical issues, which has demonstrated a higher reproducibility compared to predictive signatures based on individual genes^35–38^. A previous study combining oncogenic pathway signatures of *BRAF*, *KRAS*, and *PI3KCA* mutations revealed a favorable predictive performance of response to cetuximab for patients with colorectal cancer^37^. Another study integrated gene expression and drug sensitivity datasets measured for hundreds of anticancer compounds across 10 cancer types, and identified several pathway-based signatures which were highly predictive of cancers to therapies^35^. Unfortunately, most of published pathway-based signatures have predominantly been focused on predicting response to chemotherapy or targeted therapies^35–37^. Therefore, it warrants further investigation to construct and validate pathway-based signatures that will faithfully and accurately predict response to ICB therapies.

In this work, we develop pathway-based signatures to predict response of metastatic melanoma to anti-PD1-based therapies in four independent datasets with RNAseq data and clinical information available for both pre- and on-treatment metastatic melanomas. We identify pathway signatures that are significantly enriched in tumor specimens from anti-PD1 responders (R) compared to nonresponders (NR) at pre-treatment and on-treatment time points, respectively. We also identify pathway signatures that are differentially expressed in pre-treatment versus on-treatment samples derived from responders. Finally, we interrogate the capacity of these two types of signatures in predicting response of metastatic melanoma to anti-PD1 therapies in comparison with existing predictive signatures. Overall, we demonstrate that pathway-based signatures derived from on-treatment tumor specimens are highly predictive of response to anti-PD1 blockade therapies in patients with metastatic melanoma.

## Results

### Patient cohorts and computation framework

**Fig. 1:**
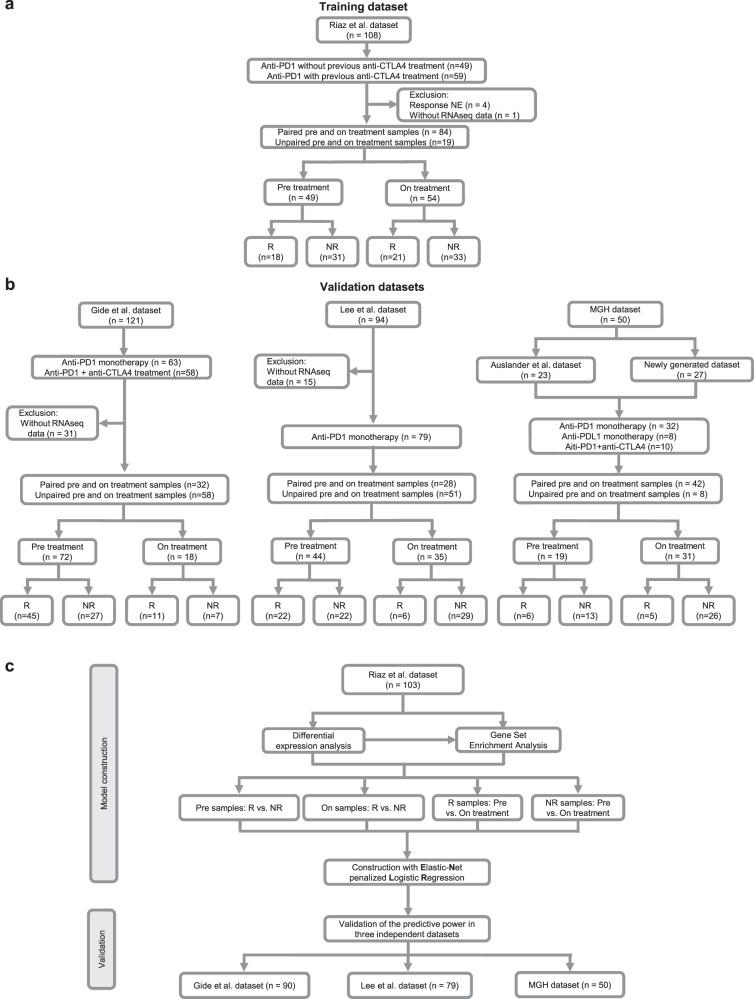
Cohort consolidation and computational workflow. **a,** **b** The flowchart of sample inclusion and exclusion criteria. **a** A melanoma cohort including patients who were treated with anti-PD-1 therapy was selected as training dataset including 49 pre-treatment and 54 on-treatment samples. **b** Three independent melanoma cohorts and one newly generated cohort were enrolled as validation datasets in this study. A total of 135 pre-treatment and 84 on-treatment samples were included for the final analysis. **c** The computational workflow of model construction and validation. Predictive models were constructed using pre-treatment and on-treatment samples, respectively, from the Riaz et al. dataset. Models were validated in three independent datasets. NE not evaluation, R responders, and NR nonresponders.

In this study, we analyzed four published datasets and one newly generated dataset with RNAseq data available for pre- and on-treatment tumor specimens derived from patients with metastatic melanoma who were treated with anti-PD-1/PD-L1 monotherapy, anti-PD-1/PD-L1 monotherapy with prior anti-CTLA-4 monotherapy, or the combination of anti-PD-1 plus anti-CTLA-4 therapies (Fig. 1a, b). We designed a computational framework to discover the pathway-based signatures, which predict patients’ response to ICB therapies. In this framework, we first generated signatures through the training dataset and subsequently tested the generalized predictive power in validation datasets. Since the sample size of Riaz et al. dataset is the largest among all four datasets, we designated it as a training dataset and assigned the other three cohorts of Lee et al., Gide et al., and the MGH cohort for the validation purpose (Fig. 1c).

In the Riaz et al. dataset^39^, 68 patients with 108 biopsies received anti-PD1 monotherapy with (59 biopsies) or without (49 biopsies) prior anti-CTLA-4 therapy (Fig. 1a and Supplementary Data 1a). After excluding those patients without response evaluation criteria in solid tumors (RECIST) (*n* = 4), and those without RNAseq data (*n* = 1), a total of 49 biopsies at the timepoint of pre-treatment and 54 biopsies at the timepoint of on-treatment were included in the analysis. This corresponded to 84 paired pre- and on-treatment biopsies and 19 unpaired biopsies. In the cohort of pre-treatment samples, 18 biopsies were from responders (R), and 31 biopsies were from nonresponders (NR). In the cohort of on-treatment samples, 21 biopsies were from R, and 33 biopsies were from NR.

In the Gide et al. dataset^40^, 54 patients with 63 biopsies received anti-PD1 monotherapy, of whom 30 patients had prior anti-CTLA4, and 51 patients with 58 biopsies were treated with the combination of anti-CTLA4 plus anti-PD1 therapies (Fig. 1b and Supplementary Data 1b). After excluding 31 biopsies without RNAseq data, we included 32 paired pre- and on-treatment biopsies and 58 unpaired biopsies in the analysis. There were 72 pre-treatment samples (45 for R and 27 for NR) and 18 on-treatment samples (11 for R and 7 for NR).

In the Lee et al. dataset^41^, 55 patients with 94 biopsies were treated with anti-PD1 monotherapy, including nivolumab (Nivo) or pembrolizumab (Pembro) (Fig. 1b and Supplementary Data 1c). A total of 28 paired pre and on-treatment biopsies and 51 unpaired biopsies were included in the analysis. There were 44 pre-treatment samples (22 for R and 22 for NR) and 35 on-treatment samples (6 for R and 29 for NR).

We also analyzed the cohort of patients with metastatic melanoma who were treated with anti-PD1/PD-L1 monotherapy at Massachusetts General Hospital (MGH), including a published dataset^14^ and a newly generated dataset for this study (Fig. 1b and Supplementary Data 1d). This MGH cohort included 42 paired pre- and on-treatment biopsies and 8 unpaired pre- and on-treatment biopsies. In the MGH cohort, there were 19 pre-treatment tumor samples (*n* = 6 for R and *n* = 13 for NR) and 31 on-treatment tumor samples (*n* = 5 for R and *n* = 26 for NR).

### Pathway-based super signature for pre-treatment samples

**Fig. 2:**
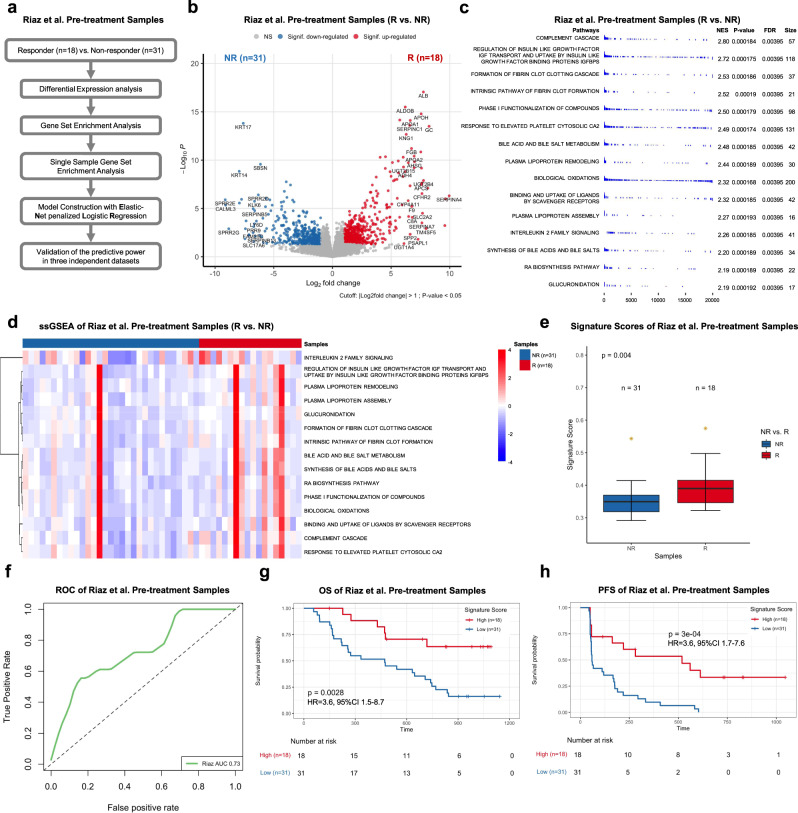
Pathway-based super signature for pre-treatment samples. **a** The computation pipeline of model construction based on pre-treatment samples in the Riaz et al. cohort. **b** The volcano plot of differential gene expression analysis between pre-treatment responders (R) and nonresponders (NR) in the Riaz et al. cohort. Log2Fold change (FC) was calculated. The two-sided Wald test was implemented to test if no differential expression between responder and nonresponders. The blue dots represent significantly downregulated (Signif. downregulated) genes (log2Fold Change < −1, *P*-value < 0.05). The red dots represent significantly upregulated (Signif. upregulated) genes (log2Fold Change > 1, *P*-value < 0.05). The gray dots represent nonsignificant (NS) genes. **c** GSEA results of 15 top ranked candidate pathways. Normalized Enrichment Score (NES) and the size of leading-edge geneset are calculated. The permutation based *P*-value shows the statistical significance of the enrichment score. The number of permutation is 10,000. False discovery rate (FDR) is the estimated probability that the normalized enrichment score represents a false positive finding. **d** The heatmap of ssGSEA values of pre-treatment responders (R) and nonresponders (NR) in the Riaz et al. cohort. Nonresponders are presented with the number of samples on the left side, and responders are presented with the number of samples on the right side. FDR-corrected two-sided Welch *t*-test was conducted to compare ssGSEA values between R and NR samples, only FDR < 0.05 showed here. **e** The boxplot of pre-treatment sample’s Pathway-based super signatures (PASS-PRE) signature score of responders (R) and nonresponders (NR) in the Riaz et al. cohort. The *P*-value was computed via a one-sided rank-sum test. Boxplot center lines indicate median, box edges represent the interquartile range, whiskers extend to the minimum and maximum, and the outliers are plotted individually using the ‘*’ symbol. **f** ROC and AUC of PASS-PRE on the pre-treatment samples in the Riaz et al. cohort. **g, h** The Kaplan–Meier survival analysis of overall survival (OS) (**g**) and progression-free survival (PFS) (**h**) of pre-treatment samples in the Riaz et al. cohort. The two-sided log-rank test compared high and low subgroups based on the mean of pre-treatment samples odd ratio as cutoff. Hazard ratio (HR) was calculated and shown with confidence interval (CI). Source data are provided as a Source Data file.

To investigate the predictive performance of signatures derived from pre-treatment samples in patients with metastatic melanoma treated with anti-PD1 blockade, we first used an Elastic-Net penalized Logistic Regression (ENLR) model to construct predictive pathway-based signatures based on pre-treatment samples in the Riaz et al. training dataset. Our pathway-based signature construction entailed a computation pipeline, which consisted of differential expression gene analysis (DEGs), geneset enrichment analysis (GSEA), filtration of candidate pathways, and training and validation of the ENLR model (Fig. 2a). By performing the analysis of DEGs, we identified 190 genes that were significantly upregulated in pre-treatment samples from R as compared to NR (Log_2_FoldChange > 1 and Wald test *p* < 0.05) (Fig. 2b and Supplementary Data 2a). To identify genesets among the MSigDB Reactome collection which were correlated with the phenotype of response in pre-treatment samples, we implemented GSEA based on the ranked gene list of DEGs and identified 98 significantly enriched pathways (enrichment score > 0 and FDR < 0.05) (Supplementary Data 2b). We ranked the filtered pathways by normalized enrichment score (NES) and focused on the top 15 ranked pathways (Fig. 2c). Next, we conducted the single-sample GSEA (ssGSEA) to derive a score for each of 15 pathways by using the leading-edge genes and compared ssGSEA values of R to those of NR by FDR-corrected Welch *t*-test (FDR < 0.05) (Fig. 2d and Supplementary Data 2c). Based on pathway scores, we subsequently employed the ENLR model to identify pathways with the highest predictive accuracy. To mitigate the imbalanced classification issue, cost-sensitivity method and three-fold cross-validate training method were implemented in the ENLR model to determine the optimized penalty parameter with the error that was within 1 standard error of the minimum and to calculate the effect size of each candidate pathway (Supplementary Fig. 1a, b). Subsequently, we obtained six pathways as the most effective features for predicting response to anti-PD1 treatment, including (1) Complement cascade; (2) Regulation of insulin like growth factor IGF transport and uptake by insulin like growth factor binding proteins IGFBPS; (3) Binding and uptake of ligands by scavenger receptors; (4) Plasma lipoprotein remodeling; (5) Interleukin 2 family signaling; and (6) RA biosynthesis pathways (Supplementary Fig. 1c, d). Complement cascade, Binding and uptake of ligands by scavenger receptors, and Interleukin 2 family signaling pathways are related to immune and inflammation, whereas Plasma lipoprotein remodeling and the RA Biosynthesis pathways are related to metabolism. Using the effective sizes as weight, we calculated a weighted average of ssGSEA values of these six pathways and named it pathway-based super signature (PASS) score. The distribution of PASS scores of pre-treatment samples (PASS-PRE) in Riaz et al. dataset demonstrated that PASS-PRE scores were significantly higher in R compared to NR (*p* = 0.004; one-sided rank-sum test) (Fig. 2e and Supplementary Data 2d). To quantify the predictive power of the PASS-PRE, we generated the Receiver Operator Characteristic (ROC) and observed an Area under the Curve (AUC) of 0.73 (Fig. 2f), highlighting a decent predictive power of PASS-PRE. Moreover, we evaluated the association of pathway signature scores with patients’ survival. We calculated each sample’s odds ratio based on the signature score, and then stratified patients into high and low subgroups by using the mean of their odds ratio as the cutoff and performed the Kaplan–Meier survival analysis for overall survival (OS) and progression-free survival (PFS). Compared to patients with low signature scores, significantly improved OS and PFS were observed in those with high signature scores (OS: HR = 3.6, 95% CI 1.5–8.7, *p* = 0.0028; PFS: HR = 3.6, 95% CI 1.7–7.6, *p* < 0.001) (Fig. 2g, h).

**Fig. 3:**
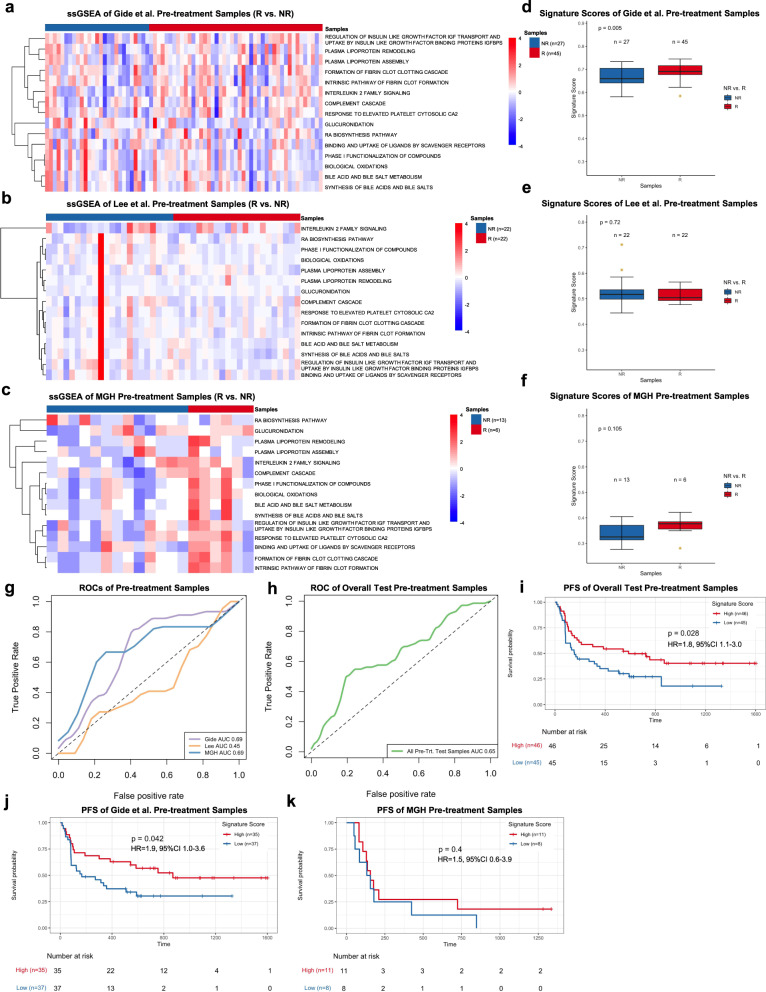
Pathway-based super signature for pre-treatment samples. **a**–**c** The heatmap of ssGSEA values of pre-treatment responders (R) and nonresponders (NR) in the Gide et al. cohort (**a**), Lee et al. cohort (**b**), and MGH cohort (**c**). Nonresponders are presented on the left side, and responders are presented on the right side. FDR-corrected two-sided Welch *t*-test was conducted to compare ssGSEA values between R and NR samples, only FDR < 0.05 showed here. **d**–**f** The boxplot of pre-treatment samples’ PASS-PRE signature scores in the Gide et al. cohort (**d**), Lee et al. cohort (**e**), and MGH cohort (**f**).The pre-treatment samples were separated into responders (R) and none-responders (NR) with number of samples showed in each cohorts. The *P*-values were computed via a one-sided rank-sum test. Boxplot center lines indicate medians, box edges represent the interquartile range, whiskers extend to the minimum and maximum, and the outliers are plotted individually using the ‘*’ symbol. **g** ROC and AUC of PASS-PRE on pre-treatment samples from Gide et al., Lee et al. and MGH cohorts. **h** ROC and AUC on the all pre-treatment test samples, which combined with Gide et al., Lee et al. and MGH cohorts. **i**–**k** The Kaplan–Meier analysis of progression-free survival (PFS) of pre-treatment samples in the combined test pre-treatment samples (**i**), Gide et al. cohort (**j**), and MGH cohort (**k**). The two-sided log-rank test compared high and low subgroups based on the mean of pre-treatment samples odd ratio as cutoff. Hazard ratio (HR) was calculated and shown with confidence interval (CI). Source data are provided as a Source Data file.

We further validated the predictive performance of PASS-PRE in three independent datasets with RNAseq data available for pre-treatment samples, including Gide et al., Lee et al., and the MGH cohort. We calculated the ssGSEA value, signature score and odds ratio for each of pre-treatment samples in each of validation datasets. We compared ssGSEA values of R to those of NR for each pathway using FDR-corrected Welch *t*-test (FDR < 0.05). The FDR result did not show a significant difference between R and NR in Gide et al., Lee et al., and the MGH dataset (Fig. 3a–c, and Supplementary Data 3a–c). The distributions of PASS-PRE scores of R in Gide et al. was significantly higher than NR (*p* = 0.005; one-sided rank-sum test) (Fig. 3d and Supplementary Data 3d). However, PASS-PRE scores of R in the Lee et al. and the MGH dataset were not significantly higher than NR (*p* = 0.72 for Lee et al.; *p* = 0.10 for MGH; one-sided rank-sum test) (Fig. 3e, f and Supplementary Data 3e, f). We generated the ROC and AUC with signature scores and AUCs were 0.69, 0.45, and 0.69 for Gide et al., Lee et al., and the MGH dataset, respectively (Fig. 3g). Additionally, we tested the predictive performance of PASS-PRE signature in two other melanoma cohorts: the Van Allen et al.^6^ and Hugo et al.^7^ datasets. The AUCs of these two datasets in pre-treatment samples were 0.56 and 0.46, respectively (Supplementary Fig. 1e). The AUCs suggested the predictive power of PASS-PRE across all six training and validation datasets was unstable. Furthermore, we combined the test pre-treatment samples of Gide et al., Lee et al., and MGH datasets and calculated the AUC with signature scores. And we generated a combined AUC of 0.65 (Fig. 3h). We used the Youden Index method to find the optimized cutoff on Riaz et al. PASS-PRE signature score, which was 0.3834. We predicted treatment response of combined test pre-treatment samples. The prediction accuracy was 0.59. And the Mathew Correlation Coefficient was 0.19. Similarly, we computed each sample’s odds ratio based on PASS-PRE signature score, and divided pre-treatment samples into two subsets with high and low signature scores using the mean value of samples’ odd ratios as a threshold. The Kaplan–Meier survival analysis of all test samples suggested that patients with higher signature scores were associated with significantly improved PFS (HR = 1.8, 95% CI 1.1–3.0; *p* = 0.028) and OS (HR = 1.9, 95% CI 1.1–3.2, *p* = 0.026) when compared to those with lower scores (Fig. 3i and Supplementary Fig. 1f). We also investigated the differences of PFS and OS for each dataset. In the Gide et al. dataset, patients with high scores were associated with better PFS (HR = 1.9, 95% CI 1.0–3.6, *p* = 0.042) but not OS (HR = 1.8, 95% CI 0.9–3.9; *p* = 0.11) (Fig. 3j and Supplementary Fig. 1g). Since PFS data were not available for the Lee et al. dataset, we only performed the OS analysis. And the results did not show a significant difference in OS between patients with high and low signature scores (HR = 1.7, 95% CI 0.5–5.1, *p* = 0.37) (Supplementary Fig. 1h). For the MGH cohort, significantly improved OS (HR = 3.4, 95% CI 1.0–12.0, *p* = 0.04) but not PFS (HR = 1.5, 95% CI 1.1–3.9, *p* = 0.4) was observed in patients with high signature scores compared to those with low scores (Fig. 3k and Supplementary Fig. 1i).

Taken together, we concluded that the predictive performance of PASS-PRE signature in the pre-treatment samples was not robust and stable enough. Lastly, six pathways selected by the ENLR model did not generalize across different datasets of pre-treatment samples.

### Pathway-based super signature for on-treatment samples

**Fig. 4:**
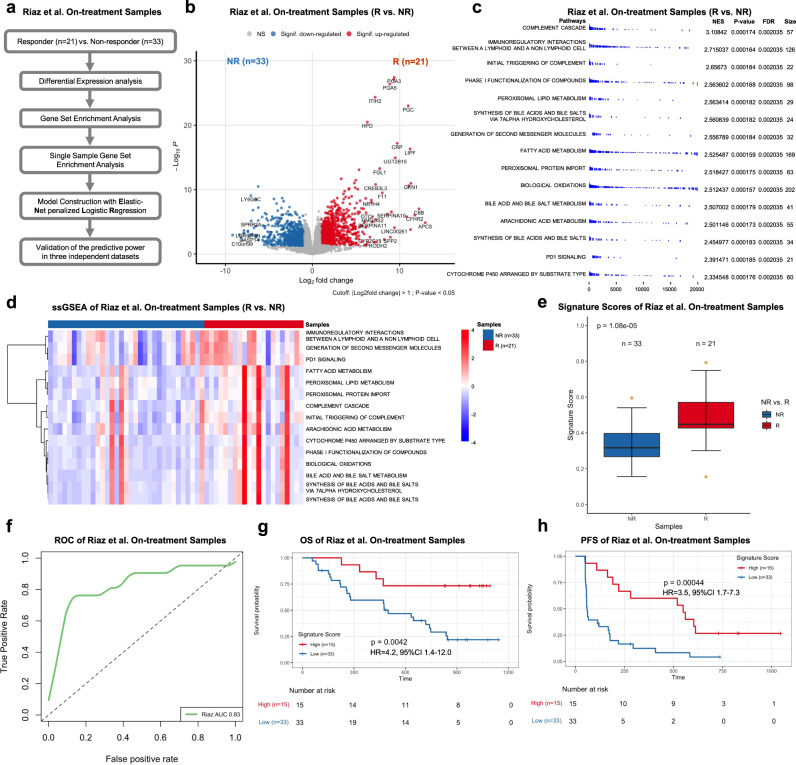
Pathway-based super signature for on-treatment samples. **a** The computational pipeline of model construction based on on-treatment samples in the Riaz et al. cohort. **b** The volcano plot of differential gene expression analysis between on-treatment responders (R) and nonresponders (NR) in the Riaz et al. cohort. Log2Fold change (FC) was calculated. The two-sided Wald test was implemented to test if no differential expression between responder and nonresponders. The blue dots represent significantly downregulated (Signif. downregulated) genes (log2Fold Change < −1, *P*-value < 0.05). The red dots represent significantly upregulated (Signif. upregulated) genes (log2Fold Change > 1, *P*-value < 0.05). The gray dots represent nonsignificant (NS) genes. **c** The GSEA results of 15 candidate pathways. Normalized Enrichment Score (NES) and the size of leading-edge geneset are calculated. The permutation based *P*-value shows the statistical significance of the enrichment score. The number of permutation is 10,000. False discovery rate (FDR) is the estimated probability that the normalized enrichment score represents a false positive finding. **d** The heatmap of ssGSEA values of on-treatment responders (R) and nonresponders(NR) in the Riaz et al. cohort. Nonresponders are presented on the left side, and responders are presented on the right side. FDR-corrected two-sided Welch *t*-test was conducted to compare ssGSEA values between R and NR samples, only FDR < 0.05 showed here. **e** The boxplot of on-treatment sample’s Pathway-based super signatures (PASS-ON) signature score of responders (R) and nonresponders (NR) with the number of samples showed in the Riaz et al. cohort. The *P*-value was computed via a one-sided rank-sum test. Boxplot center lines indicate medians, box edges represent the interquartile range, whiskers extend to the minimum and maximum, and the outliers are plotted individually using the ‘*’ symbol. **f** ROC and AUC of PASS-ON on the on-treatment samples in the Riaz et al. cohort. **g,** **h** The Kaplan–Meier analysis of overall survival (OS) (**g**) and progression-free survival (PFS) (**h**) of on-treatment samples in the Riaz et al. cohort. The two-sided log-rank test compared high and low subgroups based on the mean of on-treatment samples odd ratio as cutoff. Hazard ratio (HR) was calculated and shown with confidence interval (CI). Source data are provided as a Source Data file.

Next, we investigated the predictive performance of signatures derived from on-treatment samples. Similar to the analysis of pre-treatment samples, we constructed a computation pipeline, which consisted of DEGs, GSEA, filtration of candidate pathways, and the use of ENLR model, to derive a pathway-based signature for on-treatment samples in the Riaz et al. dataset as the training set (Fig. 4a). By performing the analysis of DEGs, we identified 1078 genes that were significantly upregulated in on-treatment samples from R as compared to NR (Log_2_FoldChange > 1 and Wald test *p* < 0.05) (Fig. 4b and Supplementary Data 4a). Subsequently, the GSEA identified 111 Reactome pathways, which were significantly enriched (enrichment score > 0 and FDR < 0.05) (Supplementary Data 4b). We ranked the filtered pathways by NES and focused on the top 15 ranked pathways as candidates for the downstream analysis (Fig. 4c). Next, we calculated ssGSEA value for each candidate pathway by using leading-edge genes and summarized it as a pathway score (Fig. 4d and Supplementary Data 4c). Based on pathway scores of 15 candidate pathways, we subsequently employed the ENLR model to identify pathways with the most predictive accuracy and estimated their effects. The ENLR model implemented both cost-sensitivity and the three-fold cross-validation in training method to determine the optimized penalty parameter with the error that was within 1 standard error of the minimum as well as calculated the effect size of each candidate pathway (Supplementary Fig. 2a, b). Consequently, a signature of four pathways was identified, including (1) Peroxisomal Lipid Metabolism; (2) Generation of Second Messenger Molecules; (3) Fatty Acid Metabolism; and (4) PD1 Signaling (Supplementary Fig. 2c, d). Of note, Peroxisomal Lipid Metabolism and Fatty Acid Metabolism are related to fatty acid and lipid metabolism^42^. Generation of Second Messenger Molecules is a pivotal signaling pathway in T-cell receptor (TCR) stimulation. PD1 Signaling plays an important role in immunoregulation as an immunoregulatory signaling pathway. Using the effective sizes as weight, we calculated a weighted average of ssGSEA values of these four pathways and named it pathway-based super signature for on-treatment sample (PASS-ON) score. The distribution of PASS-ON scores for the Riaz et al. dataset demonstrated higher scores in R compared to NR (*p* < 0.001; one-sided rank-sum test) (Fig. 4e and Supplementary Data 4d). To determine the predictive power of PASS-ON, we generated the ROC using the values of PASS-ON. We observed an AUC of 0.83, highlighting a decent predictive power of PASS-ON (Fig. 4f). We also evaluated the association between signature scores and patients’ survival. We first calculated the odds ratio based on each sample’s signature score and then stratified patients into high and low subgroups based on the mean value of the odds ratio used as the cutoff. The Kaplan–Meier survival analysis indicated that patients with high signature scores were associated with significantly longer OS and PFS compared to those with low signature scores (OS: HR = 4.2, 95% CI 1.4–12.0, *p* = 0.0042; and PFS: HR = 3.5, 95% CI 1.7–7.3, *p* = 0.00044) (Fig. 4g, h).

**Fig. 5:**
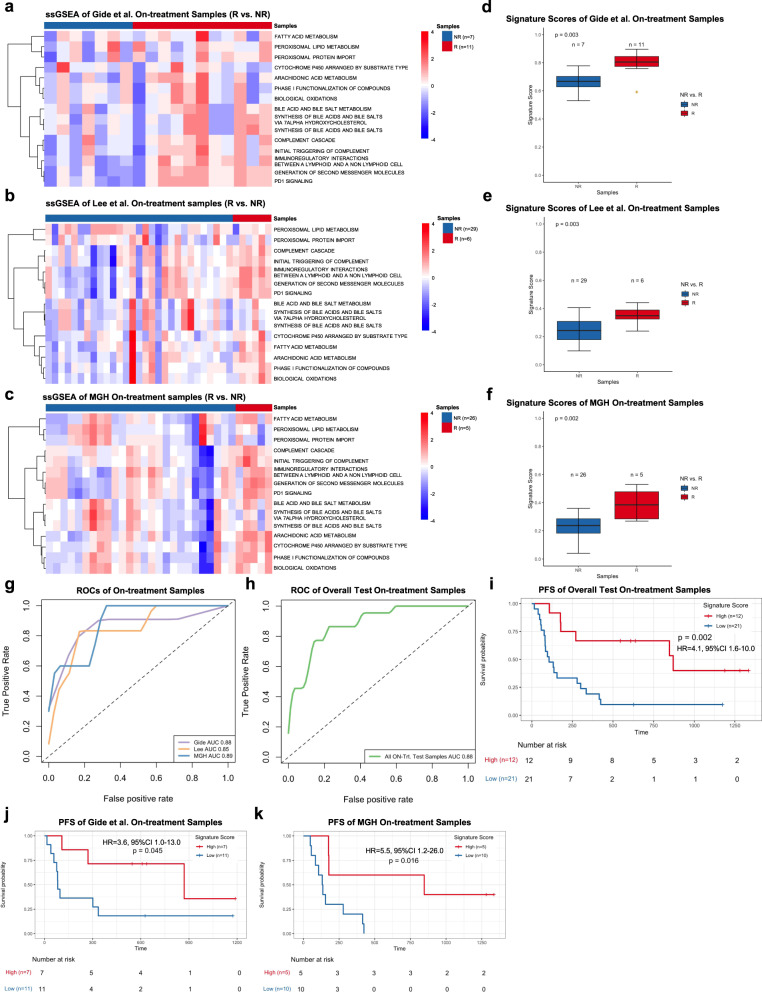
Pathway-based super signature for on-treatment samples. **a**–**c** The heatmap of ssGSEA values of on-treatment responders (R) and nonresponders (NR)in the Gide et al. cohort (**a**), Lee et al. cohort (**b**), and MGH cohort (**c**). Nonresponders are presented on the left side, and responders are presented on the right side. FDR-corrected two-sided Welch *t*-test was conducted to compare ssGSEA values between R and NR samples, only FDR < 0.05 showed here. **d**–**f** The boxplot of on-treatment samples’ PASS-ON signature scores in the Gide et al. cohort (**d**), Lee et al. cohort (**e**), and MGH cohort (**f**). The on-treatment samples were separated into responders (R) and nonresponders (NR) with number of samples showed in each cohorts. The *P-*values were computed via a one-sided rank-sum test. Boxplot center lines indicate medians, box edges represent the interquartile range, and the outliers are plotted individually using the ‘*’ symbol. **g** ROC and AUC PASS-ON on the on-treatment samples from Gide et al., Lee et al., and MGH cohorts. **h** ROC and AUC on the all on-treatment test samples which combined with Gide et al., Lee et al., and MGH cohorts. **i–k** The Kaplan–Meier analysis of progression-free survival (PFS) of on-treatment samples in the combined test on-treatment samples (**i**), Gide et al. cohorts (**j**), and MGH cohorts (**k**). The two-sided log-rank test compared high and low subgroups based on the mean of on-treatment samples’ odd ratio as cutoff. Hazard ratio (HR) was calculated and shown with confidence interval (CI). Source data are provided as a Source Data file.

To further validate the predictive performance of PASS-ON, we tested three independent datasets with RNAseq data available for on-treatment samples including Gide et al., Lee et al., and the MGH cohort. We calculated ssGSEA values and compared ssGSEA values between R and NR for each pathway (FDR < 0.05; FDR-corrected Welch *t*-test) (Fig. 5a–c, and Supplementary Data 5a–c). We also calculated the signature score and odds ratio for each on-treatment sample in validation datasets. Similar to the Riaz et al. dataset, the distribution of signature scores derived from on-treatment samples demonstrated that the signature scores of R were significantly higher than those of NR in the Gide et al. (*p* = 0.003), Lee et al. (*p* = 0.003), and MGH datasets (*p* = 0.002) (Fig. 5d–f, and Supplementary Data 5d–f), respectively. We generated the ROC and AUC with signature scores and patients’ response data. The AUCs were 0.88, 0.85, and 0.89 for Gide et al., Lee et al., and MGH datasets, respectively, which suggested a stable and acceptable predictive power of PASS-ON across all four datasets (Fig. 5g). We also combined all on-treatment samples from Gide et al., Lee et al., and MGH test datasets and generated an AUC of 0.88 (Fig. 5h). Further, we implemented the Youden Index method to derive the optimized cutoff, which was 0.4247, from Riaz et al. on-treatment sample PASS-ON signature scores. Using the optimized cutoff, we calculated the accuracy of the PASS-ON signature on the combined test samples. The accuracy was 0.82 and the Mathew Correlation Coefficient was 0.53. Next, we divided on-treatment samples into high and low subsets by using the mean value as a threshold based on the odds ratio and performed the Kaplan–Meier survival analysis in each individual or combined datasets. Patients with high signature scores were associated with significantly improved PFS compared to those with low signature scores in all test patients (HR = 4.1, 95% CI 1.6–10.0, *p* = 0.002), Gide et al. (HR = 3.6, 95% CI 1.0–13.0, *p* = 0.045), and MGH datasets (HR = 5.5, 95% CI 1.2–26.0, *p* = 0.016) (Fig. 5i–k). Significantly higher OS was also observed in patients with higher signature scores in the MGH cohort (HR = 5.6, 95% CI 1.1–28.0, *p* = 0.02) but not all test patients (HR = 1.8, 95% CI 0.8–4.1, *p* = 0.14), Gide et al. (HR = 2.7, 95% CI 0.5–14.0, *p* = 0.23), or Lee et al. (HR = 0.65, 95% CI 0.2–2.4, *p* = 0.52) cohorts (Supplementary Fig. 2e–h).

From the analysis of on-treatment samples, we identified and demonstrated PASS-ON effectiveness in predicting patients’ clinical response to anti-PD1 therapies. Furthermore, PASS-ON scores were able to distinguish patients with better survival outcomes from those with worse ones.

### Time-response interaction pathway-based super signatures for pre- and on-treatment samples

**Fig. 6:**
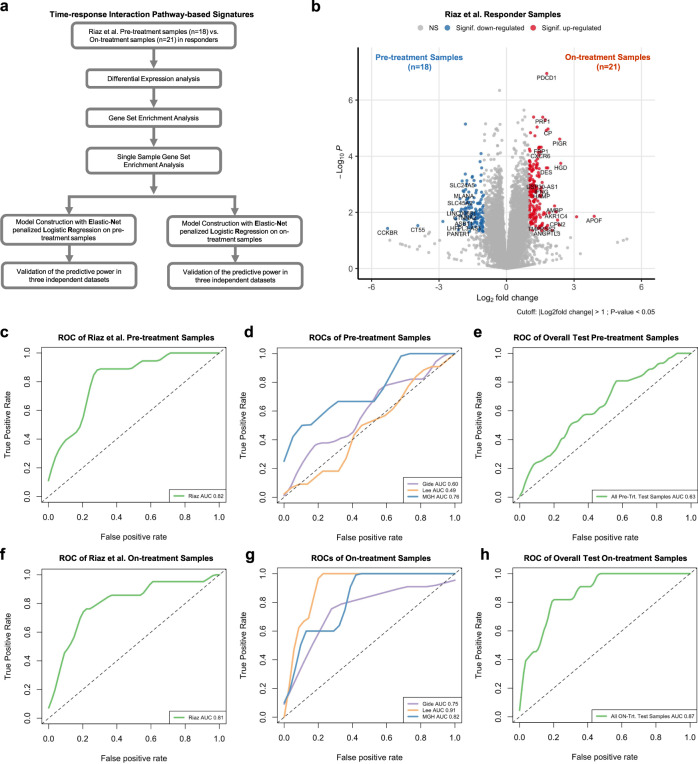
Time-response interaction pathway-based super signatures for pre- and on-treatment samples. **a** Computation pipeline of model construction of time-response interaction pathway-based Super Signatures. **b** The volcano plot of differential gene expression analysis of response pre-treatment and on-treatment samples in the Riaz et al. cohort. Log2Fold change (FC) was calculated. The two-sided Wald test was implemented to test if no differential expression between response pre-treatment and on-treatment samples. The blue dots represent significantly downregulated (Signif. downregulated) genes (log2Fold Change < −1, *P*-value < 0.05). The red dots represent significantly upregulated (Signif. upregulated) genes (log2Fold Change > 1, *P*-value < 0.05). The gray dots represent nonsignificant (NS) genes. **c–e** ROCs and AUCs of the time-response interaction pathway-based super signatures for pre-treatment samples (TimeANLS-PRE) from Riaz et al. cohort (**c**), Gide et al., Lee et al., and MGH cohorts (**d**), combined pre-treatment with Gide et al., Lee et al., and MGH cohorts (**e**). **f**–**h** ROCs and AUCs of the time-respons**e** interaction pathway-based super signatures for on-treatment samples (TimeANLS-ON) from Riaz et al. cohort (**f**), Gide et al., Lee et al., and MGH cohorts (**g**), combined on-treatment with Gide et al., Lee et al., and MGH cohorts (**h**). Source data are provided as a Source Data file.

In contrast to pathway-based super signatures which were fundamentally based on the comparison between R and NR samples at pre- or on-treatment timepoint, we were also interested in investigating the predictive performance of pathway-based signatures that reflect the time-response interaction. We reasoned that those signatures were dynamically changed during the treatment. In the analysis of identifying pathway-based signatures that reflect the time-response interaction, we implemented two independent ENLR models to derive predictive signatures from pre-treatment and on-treatment samples in Riaz et al. dataset, respectively. We constructed the computation pipeline, which consisted of DEGs, GSEA, and candidate pathways filtration. It is worth noting that the two independent ENLR models shared the same candidate pathways. Two independent ENLR models in the pipeline were then implemented to calculate the ssGSEA values and to derive pathway-based signatures from pre-treatment and on-treatment samples, respectively (Fig. 6a). Specifically, we performed DEGs to identify genes whose expression levels were changed between the pre-treatment and on-treatment timepoint in R samples in the Riaz et al. dataset (unpaired analysis). The volcano plot showed 60 upregulated genes in R samples (Log_2_FoldChange > 1 and Wald test *p* < 0.05) (Fig. 6b and Supplementary Data 6a). We further performed GSEA by using the ranked gene list and identified 110 significantly upregulated pathways. We selected the top 15 ranked pathways in R samples as candidates (Supplementary Fig. 3a and Supplementary Data 6b). The pre-treatment samples’ ssGSEA values were calculated for each candidate pathway and summarized as a pathway score. Based on the FDR-corrected Welch *t*-test, the pathway scores did not show an obvious difference between R and NR in pre-treatment samples from all datasets (Supplementary Fig. 3b-e and Supplementary Data 7a–d). We employed the first independent ENLR model to screen the predictive pathways from the 15 candidates and estimated their effects. The three-folder cross-validate method was implemented to determine the number of pathways with associated effective sizes in the training process using the first independent ENLR model (TimeANLS-PRE) (Supplementary Fig. 3f-h). Using the effective sizes as weight, we calculated a time-interaction pathway-based super signature score for each pre-treatment sample. From the distributions of signature scores in the Riaz et al., Gide et al., Lee et al., and MGH datasets, R samples generally have higher values than NR (Supplementary Fig. 3i-l and Supplementary Data 8a–d). To quantify the predictive power of the super signature in both training and validation datasets, we generated the ROC. We observed an AUC of 0.82 in the Riaz et al. dataset (Fig. 6c), and AUCs of 0.60, 0.49, and 0.76 in the Gide et al., Lee et al., and MGH datasets, respectively (Fig. 6d). We also combined all test pre-treatment samples and generated an AUC of 0.63 (Fig. 6e). Additionally, the AUC with TimeANLS-PRE signature on Van Allen et al. and Hugo et al. pre-treatment samples were 0.59 and 0.76, respectively (Supplementary Fig. 3m). The ROC and AUC results in training and validation datasets suggested our time-response interaction pathway-based super signature was not stable based on pre-treatment samples. We used the mean value of the odds ratio, which was derived from signature scores, as the cutoff to stratify patients into high and low groups. We evaluated the association between signature scores and patients’ survival, which showed significantly improved OS (HR = 2.4, 95% CI 1.1–5.3, *p* = 0.03) but not PFS (HR = 1.6, 95% CI 0.9–3.1, *p* = 0.13) was observed in patients with high signature scores when compared to those with low signature scores in Riaz et al. dataset (Supplementary Fig. 4a, b). No significant differences of neither OS nor PFS between patients with high and low signature scores were observed in overall test pre-treatment cohort, Gide et al., Lee et al., or MGH cohorts (Supplementary Fig. 4c–h).

For on-treatment samples, we tested the difference of ssGSEA values between R and NR in on-treatment samples from all datasets (FDR < 0.05; FDR-corrected Welch *t*-test) (Supplementary Fig. 5a–d and Supplementary Data 9a–d). We employed the second independent ENLR model (TimeANLS-ON) to screen the predictive pathways from the 15 candidates and estimated their effects. The TimeANLS-ON model demonstrated a similar profile as the TimeANLS-PRE model, also adopting the three-folder cross-validate method in the training process (Supplementary Fig. 5e–g). The distribution of signature scores showed that R samples had higher value than NR samples in the Riaz et al., Gide et al., Lee et al., and MGH datasets (Supplementary Fig. 5h–k and Supplementary Data 10a–d). To quantify the predictive power of the super signature in both training and validation datasets, we generated the ROC. We observed an AUC of 0.81 in the Riaz et al. dataset (Fig. 6f), and AUCs of 0.75, 0.91, and 0.82 in the Gide et al., Lee et al., and MGH datasets, respectively (Fig. 6g). We also combined all test on-treatment samples and generated an AUC of 0.87 (Fig. 6h). The ROC and AUC results in training and validation datasets suggested the time-response interaction pathway-based super signature had a decent predictive power for on-treatment samples. We also used the mean value of the odds ratio which was derived from on-treatment sample’s signature score as the cutoff to stratify samples into high and low groups. When compared to patients with low signature scores, those with high signature scores were associated with significantly improved both OS (HR = 3.7, 95% CI 1.6–8.7, *p* = 0.0016) and PFS (HR = 4.6, 95% CI 2.1–9.8, *p* < 0.0001) in the Riaz et al. (Supplementary Fig. 6a, b). However, no significant differences in OS analysis were observed in the overall test for on-treatment cohorts, Gide et al. and Lee et al. (Supplementary Fig. 6c–g). In the MGH dataset, patients with high signature scores were associated with better OS (HR = 5.6, 95% CI 1.1–28.0, *p* = 0.02) but not PFS (HR = 2.7, 95% CI 0.8–9.3, *p* = 0.11) compared to those with low signature scores (Supplementary Fig. 6h, i).

### Compare pathway-based super signatures with published predictive signatures

We further interrogated the predictive performance of PASS-PRE and PASS-ON signatures, respectively, in comparison with that of existing transcriptome-based predictive signatures, including IFN-γ signature^17^, T-cell-inflamed GEP^17^, Chemokine signature^10^, Immunoscore^10^, cytolytic activity (CYT)^13^, MHC-I^19^, MHC-II^19^, CD8A/CSF1R ratio^43^, CD8^+^ T cells CIBERSORT^44^, and IMPRES^14^. We derived the AUC of each published predictive signature across four datasets and then calculated the average AUC.

**Fig. 7:**
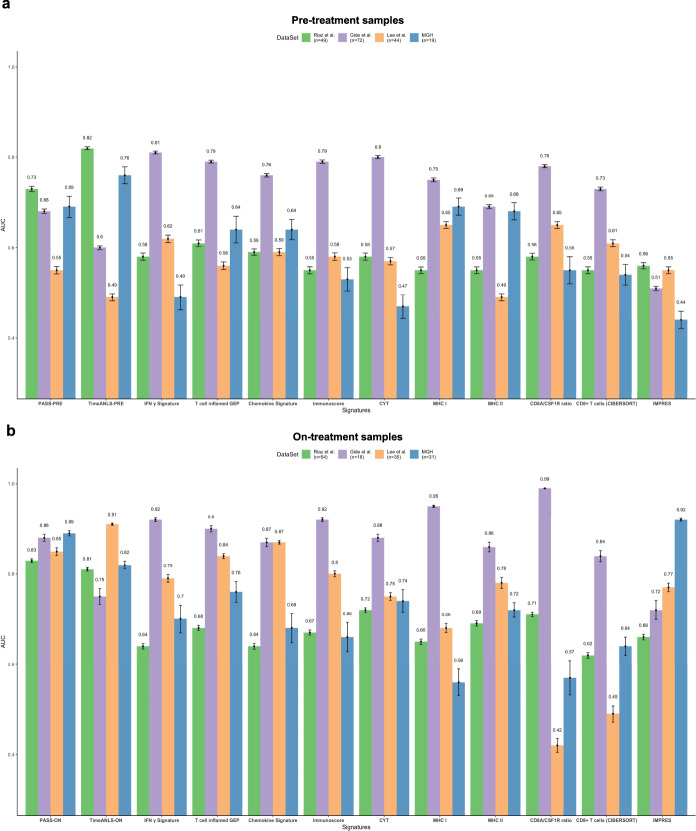
The comparison of pathway-based super signatures with existing signatures. **a** The performance of pathway-based super signature (PASS-PRE) and time-response interaction pathway-based super signatures for pre-treatment samples (TimeANLS-PRE) in comparison with publicly existing signatures. **b** The performance of pathway-based super signature (PASS-ON) and time-response interaction pathway-based super signatures for on-treatment samples (TimeANLS-ON) with existing signatures. Each cohort’s sample number were showed in the legend. The mean value and standard deviation of AUCs were showed as error bar. Source data are provided as a Source Data file.

First, we compared the predictive performance of PASS-PRE and TimeANLS-PRE with those of published predictive signatures by analyzing on pre-treatment samples. PASS-PRE and TimeANLS-PRE showed average AUCs of 0.66 and 0.67, respectively, which was comparable to other published predictive signatures (AUCs ranging from 0.51 to 0.66) (Fig. 7a and Supplementary Fig. 7a).

Similarly, we compared the predictive performance of PASS-ON and TimeANLS-ON with that of published signatures. PASS-ON and TimeANLS-ON achieved average AUCs of 0.86 and 0.82, respectively, which were superior to any other published signatures (AUCs ranging from 0.65 to 0.80) (Fig. 7b and Supplementary Fig. 7b).

## Discussion

Although ICB therapies have revolutionized treatment of metastatic melanoma, only a subset of patients has achieved a durable response^1–4^. To counteract considerable side effects and costs of ICB therapies, the identification of robust signatures predictive of response to ICB therapies is warranted in order to inform and optimize therapeutic decisions. Previous studies have reported genomic and immune signatures that predict the response of metastatic melanoma to ICB therapies^5–19,28,45^; however, most of those signatures were exclusively constructed based only on pre-treatment tumor samples or peripheral blood specimens. Furthermore, predictive signatures were generally derived from single genes or genesets of a limited number of genes. Therefore, the desire remains to identify pathway-based signatures that predict response to ICB therapies. Herein, we have filled that knowledge gap by analyzing both pre- and on-treatment tumor samples and developing pathway signatures to predict response of metastatic melanoma to anti-PD1 therapy in four published cohorts with RNAseq data and one newly generated cohort with RNAseq data. We found that pathway-based signatures derived from on-treatment tumor specimens are not only predictive of response to anti-PD1 blockade in patients with metastatic melanoma but also associated with disease outcomes.

Previous studies have suggested that on-treatment tumor samples may be more informative compared to pre-treatment samples with regards to predicting response of breast cancer to endocrine therapy^46–48^. Moreover, predictive signatures derived from on-treatment samples in predicting response of breast cancer to endocrine therapy are superior to those derived from pre-treatment samples^46,47^. To date, however, gene expression signatures that predict response to ICB therapies have largely been limited to investigating the association between pre-treatment samples and patients’ clinical response. In contrast, Chen et al. previously developed adaptive immune signatures based on tumor samples obtained during the early course of treatment, and showed that they were highly predictive of response to ICB therapies in patients with metastatic melanoma^8^. However, they performed gene expression profiling via a custom 795-gene NanoString panel rather than whole-transcriptomic sequencing. Moreover, they did not evaluated the predictive capacity of their signatures in other independent datasets. Auslander et al. built an immuno-predictive score (IMPRES), which encompasses 15 pairwise transcriptomic relations between immune checkpoint genes to predict response of metastatic melanoma to ICB therapy^14^. The IMPRES signature has showed higher predictive capacity with on-treatment samples compared to pre-treatment samples in two independent datasets^14^. This signature, however, was initially built from neuroblastomas. In this study, we developed pathway signatures based on pre-treatment and on-treatment metastatic melanomas, respectively, and exclusively, to predict response to anti-PD1 therapy in four independent datasets in which RNAseq and clinical data are available. We found that pathway-based signatures derived from on-treatment tumor specimens improved accuracy of predicting response to anti-PD1 blockade and disease outcomes in patients with metastatic melanoma when compared to those derived from pre-treatment samples. This indicated that the identification and evaluation of signatures should be attempted not only in pre-treatment samples but more importantly in tumor samples once ICB therapies have been initiated. This makes sense from the biological standpoint of view since cancer therapies generally induce complicated changes in genes and signaling pathways. Therefore, on-treatment samples may provide more valuable insights and a window into dynamic changes at the transcriptional level that is correlated with clinical response, thus resulting in higher predictive accuracy. These findings also have vital clinical implications and will lead us to reconsider clinical management in the current clinical practice, as only pre-treatment samples were profiled from most patients who received ICB therapies in previous studies. Based on these on-treatment signatures, medical oncologists can accurately predict and potentially identify this subgroup of patients who will more likely benefit from ICB therapies. For those who may not benefit from anti-PD1 therapy, other effective therapeutics such as targeted therapies tailored to tumors’ genetic composition can be administrated as early as possible. This would reduce the disease burden and incidence of side effects caused by ICB therapies and result in better disease outcomes.

In addition to using on-treatment samples, we also employed pathway-based methods to build predictive signatures. Actually, pathway-based methods have already been introduced to build predictive signatures of response to chemotherapy or targeted therapies, which showed higher reproducibility and more robust predictive performance than signatures on individual gene level^35–38^. Again, from a biological standpoint, this makes sense as cancer therapies generally alter numerous genes simultaneously; thus, pathway-based methods would make it possible to not only integrate readouts across multiple genes and genesets but also generate a more stable metric to mitigate adverse effect because of the noisy expression pattern of individual genes^35,38^. In this study, pathway signatures showed more stable predictive performances of response to anti-PD1 therapies across different cohorts of metastatic melanoma compared to existing predictive signatures for on-treatment samples. These findings suggest that pathway-based methods could help reduce effects that are unique to sequencing and profiling platforms and batches across different datasets, resulting in predictive signatures that are highly robust and reproducible. Furthermore, pathway-based methods could also help us identify previously neglected pathways, which are significantly altered by cancer therapies, but are otherwise unable to be recognized at single-gene expression level. In the present study, we identified four pathways including (1) Peroxisomal Lipid Metabolism; (2) Fatty Acid Metabolism; (3) Generation of Second Messenger Molecules; and (4) PD1 Signaling to construct the predictive model tailored to on-treatment samples. Interestingly, two of them were related to fatty acid and lipid metabolism. To our best knowledge, this is the first study revealing the predictive capacity of fatty acid and lipid metabolism-related signatures in terms of response to ICB therapies in patients with metastatic melanoma. A recent study profiled the proteome of samples from patients with metastatic melanoma undergoing either tumor infiltrating lymphocyte-based or anti-PD1 immunotherapy, and revealed that fatty acid oxidation pathway was significantly enriched in responders^42^. This study highly emphasized the important role of mitochondrial metabolism including fatty acid metabolism in conferring response to immunotherapy^42^. Theoretically, higher mitochondrial activity in tumor from responders might consume less glucose when compared to nonresponders, which reduce the glucose competition with cytotoxic T lymphocytes and thereby result in response to immunotherapy^42^. Other reports also indicated that promoting fatty acid catabolism can improve the cytotoxic ability of CD8^+^ T lymphocytes and slow down tumor progression upon treatment with immunotherapy^49,50^. The findings from these studies along with our own findings provide a biologically solid foundation for metabolism-related signatures to predict response to immunotherapies, which warrant further mechanistic investigation.

In addition to pathway-based super signature PASS-ON that was interrogated for the comparison between R and NR samples, we also investigated the predictive performance of pathway-based signatures that reflect time-response interaction. Taking the interaction of treatment time and clinical response into consideration would provide a unique insight into identifying predictive signatures that were dynamically changed during the treatment. In this study, we built two ENLR models based on time-response interaction analysis and performed the prediction using pre-treatment and on-treatment samples, respectively. Signatures across three datasets achieved AUCs of 0.49–0.76 for pre-treatment samples (Fig. 6d). Similarly, signatures across three datasets achieved AUCs of 0.75–0.91 for on-treatment samples (Fig. 6g). Taken together, these results indicated that predictive performance from on-treatment samples is more robust and superior to that derived from pre-treatment samples.

When compared to previously published signatures, PASS-ON signature showed more robust and stable predictive performance (Fig. 7b). As discussed above, on-treatment tumor specimens are generally much more informative compared to pre-treatment specimens; and pathway-based signatures can overcome limitations of individual gene-based signatures. These advances might explain, to a certain extent, why PASS-ON signature is superior to previously published signatures, which are derived from pre-treatment tumor specimens. Even though we have demonstrated the strength and superior performance of PASS-ON signature, several limitations of this signature still exist, which remain to be further refined. First, because of the limitation of dataset access, only four cohorts of metastatic melanoma were analyzed in this study. It will be critically important to further validate the predictive performance of PASS-ON signature in other independent cohorts before it can be implemented for clinical use. Secondly, PASS-ON signature was generated and validated in anti-PD1/PD-L1 based cohorts but had not been validated in anti-CTLA4-based cohorts. Furthermore, it will be interesting to test the predictive capacity of this signature in other types of ICB therapies such as CAR-T or oncolytic viral therapies. Lastly, PASS-ON signature was only validated in cohorts of metastatic melanoma. Therefore, further studies are warranted to test the predictive performance of this signature in other types of cancers.

In summary, pathway signatures derived from on-treatment samples are highly predictive of therapeutic response to anti-PD1 therapy in patients with metastatic melanoma. Importantly, we have shown their robust and stable predictive performance across different cohorts of metastatic melanoma. Our study not only provides highly accurate and personalized predictive signatures of response to anti-PD1 therapies but also sheds light on the clinical management of patients with metastatic melanoma treated with anti-PD1 therapy. Further studies are warranted to validate the predictive performance of these signatures in larger cohorts of patients with metastatic melanoma, other types of ICB therapies and cancers.

## Methods

### Melanoma datasets and patient selection

Collectively, we analyzed three published melanoma datasets, Riaz et al.(GEO accession number GSE91061), Gide et al. (BioProject accession number PRJEB23709), Lee et al. (EGA accession number EGAD00001005738), a published MGH cohort (GEO accession number GSE115821), and the newly generated MGH cohort (GEO accession number GSE168204), in which patients with metastatic melanoma were treated with anti-PD1 therapy and both pre-treatment and on-treatment biopsy samples were subject to RNA sequencing. For the newly generated cohort, patient samples were collected under the Institutional Review Board (IRB) protocols of Dana-Farber Cancer Institute (protocol 11–181) and The Wistar Institute (Human subjects protocol 2802240). Written informed consent was obtained from each patient. We also analyzed two other melanoma cohorts with only pre-treatment samples, Van Allen et al. (dbGaP accession number: phs000452.v2.p1) and Hugo et al. (GEO accession number GSE78220) cohorts. For this study, those patients’ tumor specimens, which were not subject to RNA sequencing, lacked response evaluation, or those patients with duplicated tumor specimens of the same timepoint, were excluded for further analysis. Patients’ responses to anti-PD1 therapy were assessed according to RECIST criteria. Responders were defined as patients with complete response (CR), partial response (PR), or stable disease (SD) with progression-free survival (PFS) longer than 180 days; and nonresponders were defined as progressive disease (PD) or SD with PFS shorter than 180 days.

### RNA sequencing and data processing

In the newly generated MGH cohort, a total of 27 pre- and on-treatment tumor specimens derived from metastatic melanoma patients with anti-PD1/PD-L1 treatment were used for RNA sequencing (RNAseq). The methods of RNAseq have been described previously^51^. Briefly, RNA was extracted from fresh frozen tumors by use of the Qiagen RNeasy Mini kit. RNA libraries were prepared with 250 ng RNA per sample following the standard Illumina protocols. RNAseq was performed at the Broad Institute (Illumina HiSeq2000) and the Wistar Institute (Illumina NextSeq 500). After sequencing, paired fastq files were aligned to GRCh37 reference genome by star^52^ with default settings. After obtaining the BAM files, read counts were summarized by featureCounts^53^ with the setting that only paired-ended, not chimeric and high qualified (mapping quality ≥ 20) reads were counted. R package edgeR^54^ was applied to eliminate the bias of sequencing depths and gene lengths, and RPKMs (Reads Per Kilobase of transcript per Million mapped reads) were generated.

### Differentially expressed gene analysis

Differential expression analysis used DESeq2, an R-package. The DESeq2 profiles genes according to model gene count expression data^55^ and calculates Log2Fold Change, which estimates the effect size and represents gene changes between comparison groups. The two-sided Wald-test statistics are computed to exam the differential expression across the two sample groups. And the *p*-values are corrected by multiple testing using FDR/Benjamini–Hochberg (BH) method. We used the volcano plots to visualize the differential gene expression results.

### Geneset enrichment analysis (GSEA)

Geneset enrichment analysis (GSEA) was implemented by using fgsea, an R-package. The fgsea used both the preranked gene list that was attained based on differential expression result and the Reactome geneset (Version 7.1), which is the curated geneset stored on Molecular Signature Database. The permutations *p*-value was 10,000. We filtered fgsea result based on the criteria that the pathway’s enrichment score > 0 and adjust *p*-value < 0.05. We selected the top 15 pathways as candidates based on the normalized enrichment score from the filtered list. The selected pathways results were visualized on the GSEA table.

### Single-sample GSEA (ssGSEA)

We normalized the RNAseq gene raw counts to transcripts per kilobase million (TPM) expression values using GENCODE version 35 as the reference transcript database. The R-package, GSVA^56^ computed the single-sample GSEA (ssGSEA) value of each selected pathways. The heatmap was used to visualize the ssGSEA values with Hierarchical clustering by using the “Pearson correlation” method to measure the distance and the “Complete” method for the clustering approach. Each row represented a specific pathway, and the column represented samples.

### Signature score calculation

Elastic-Net penalized logistic regression model was used to determine the most prognostic signatures from the candidate pathways. With the ssGSEA values as input, the R-package glmnet fits the penalized logistic regression model, which conducts the three-folder cross-validation in the model training process to avoid overfitting^57^. We used Cost-Sensitive method to conquer the imbalanced classification problem, which is the offset in our penalized logistic regression model. The receiver operator characteristic (ROC) curve was plotted to visualize prediction performance. The area under the curve (AUC) was calculated for each curve to quantifying signature’s prediction power. We found the optimized threshold from Riaz et al. training samples’ signature score by using Youden index method (48). We combined all the test samples from Gide et al., Lee et al., and MGH datasets. We calculated both accuracy (range from 0 to 1) and Mathew Correlation Coefficient (range from −1 to 1) on all test samples. At final, we calculated the sample’s odd ratio based on the signature score.

### Comparing our signatures predictive performance to other published signatures

We compared the performance of our signatures to other published signatures. To calculate the *P-*value, we perform 1000 repetitions of: (i) randomly sampling 80% of the samples in a stratified manner and maintaining the proportion of R versus NR for each cohort, (ii) evaluating the AUC resulting from each predictor on the randomly selected samples. (iii) implementing one-sided rank-sum test between our signatures’ AUCs and other published signatures’ AUCs.

### Statistical analysis

FDR-corrected two-sided Welch *t*-test was conducted to compare ssGSEA values between R and NR samples. One-sided rank-sum test was conducted to compare the signature scores between R and NR. Survival analysis was conducted with the Kaplan–Meier method, and samples were separated into high and low group use mean value of samples’ odd ratio as cutoff. The two-sided log-rank test was used to determine statistical significance. Hazard ratio (HR) was calculated using cox regression, while we found that the proportional Hazards assumption generally held well in most datasets. One-sided rank-sum test was conducted to compare the prediction power between our signatures and other published signatures. Each time randomly selected 80% responders and nonresponders samples from each cohort and calculate each signature;s AUC value. The process was repeated 1000 times.

### Reporting summary

Further information on research design is available in the Nature Research Reporting Summary linked to this article.

## Supplementary information

Supplementary Information

Description of Additional Supplementary Files

Supplementary Data 1

Supplementary Data 2

Supplementary Data 3

Supplementary Data 4

Supplementary Data 5

Supplementary Data 6

Supplementary Data 7

Supplementary Data 8

Supplementary Data 9

Supplementary Data 10

Reporting Summary

## Data Availability

The transcriptomic data of the newly MGH patients generated in this study have been deposited in the GEO database under accession code GSE168204. All patients’ data analyzed from published papers are referenced to and publicly available accordingly. The Riaz et al. data used in this study are available in the GEO database under accession code GSE91061. The Gide et al. data used in this study are available in the BioProject database under accession code PRJEB23709. The Lee et al. data used in this study are available in the EGA database under accession code EGAD00001005738. The published MGH data used in this study are available in the GEO database under accession code GSE115821. The Hugo et al. data used in this study are available in the GSE database under accession code GSE78220. The access to the Van Allen et al. dataset under the accession number phs000452.v2.p1 was obtained after the data access request was approved [https://www.ncbi.nlm.nih.gov/projects/gap/cgi-bin/study.cgi?study_id=phs000452.v2.p1]. Source data are provided with this paper.

## Source data

Source data

## Code availability

Codes are implemented in R and are publicly available in GitHub^58^: https://github.com/dukekuang/PASS-ON-codes.
