# Supplementary material for: Pathway signatures derived from on-treatment tumor specimens predict response to anti-PD1 blockade in metastatic melanoma
Source: Nat Commun. 2021 Oct 15;12:6023. doi: 10.1038/s41467-021-26299-4 (PMC8519947; doi:10.1038/s41467-021-26299-4)
Supplement: Supplementary file 1 — Supplementary Information [file 41467_2021_26299_MOESM1_ESM.pdf]

Supplementary Figure 1

a

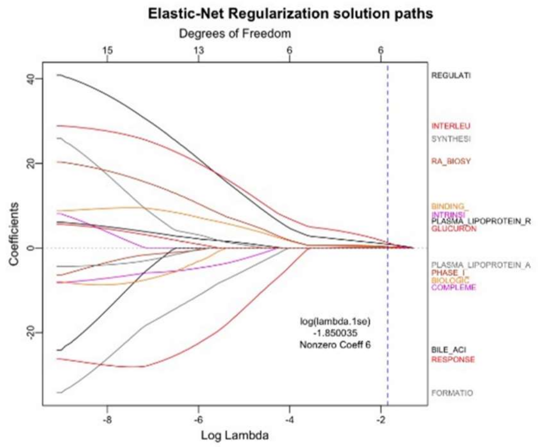

b

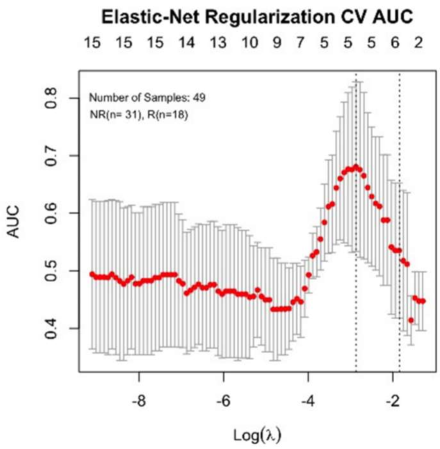

c

PASS-PRE Model Effect Size Of Candidate Pathway

Pathway	Weight
Intercept	0.35412
COMPLEMENT CASCADE	0.19999
REGULATION OF INSULIN LIKE GROWTH FACTOR IGF TRANSPORT AND UPTAKE BY INSULIN LIKE GROWTH FACTOR BINDING PROTEINS IGFBPS	0.88312
BINDING AND UPTAKE OF LIGANDS BY SCAVENGER RECEPTORS	0.17182
PLASMA LIPOPROTEIN ASSEMBLY	0.0204
INTERLEUKIN 2 FAMILY SIGNALING	1.24241
RA BIOSYNTHESIS PATHWAY	0.28956

Only shows pathways which effect size is not zero

d

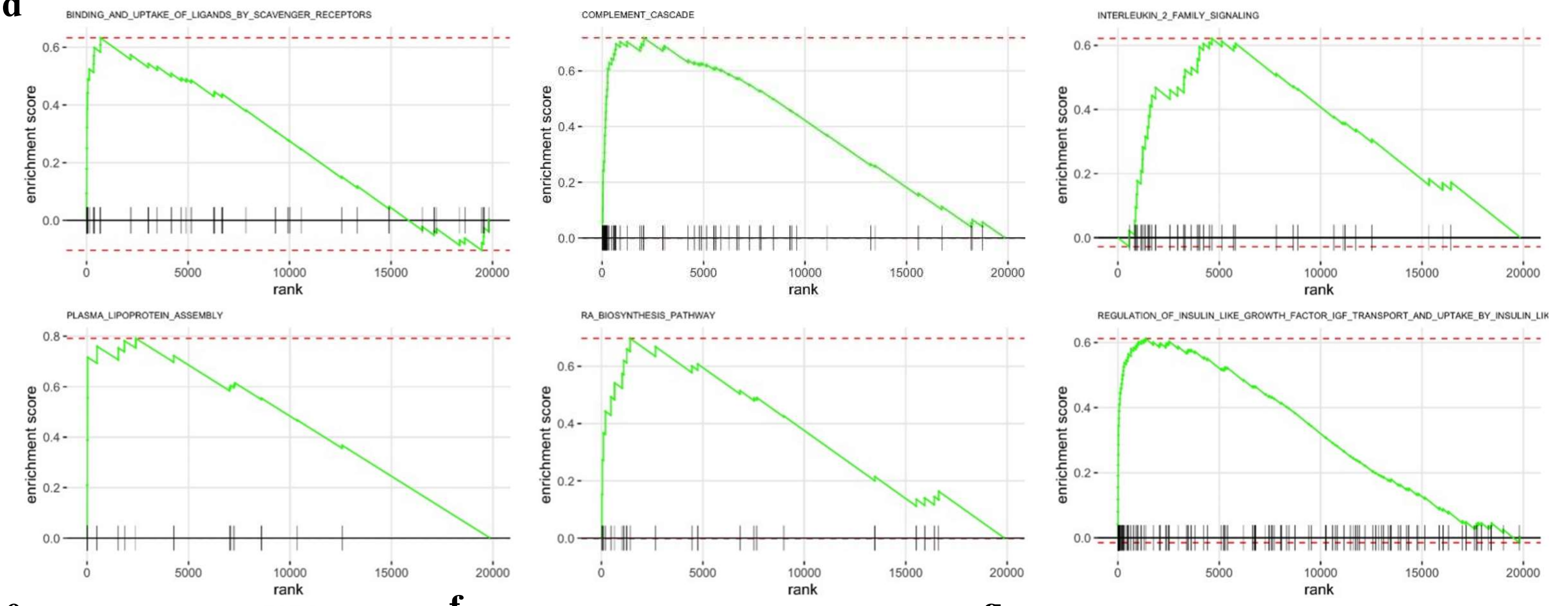

e

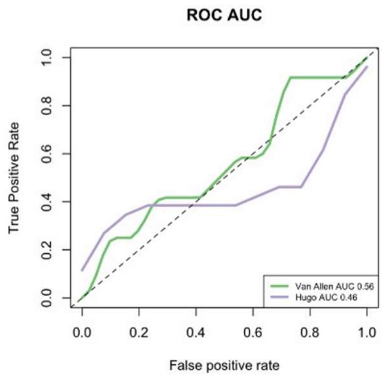

f

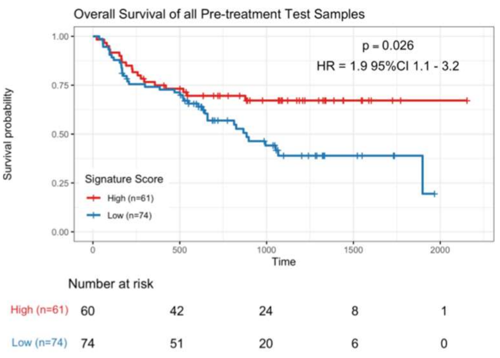

g

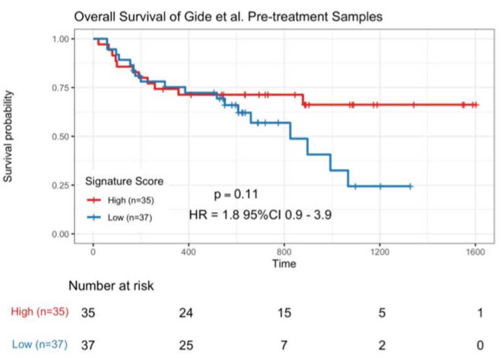

h

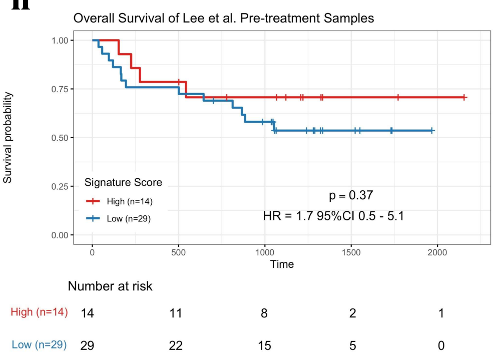

i

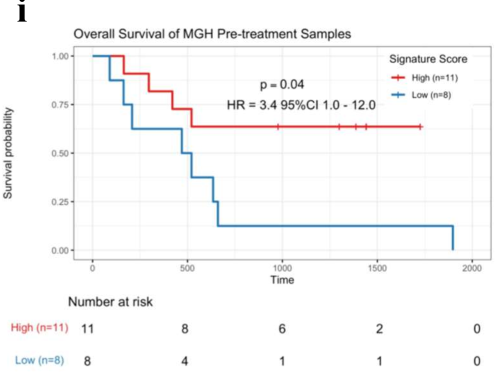

Supplementary Figure 1. Pathway-based Super Signature for Pre-treatment Samples. (**a, b**) The model's training parameter selection process on Riaz et al. pre-treatment samples to generate PASS-PRE signature, and each candidate pathways selection path associate with coefficient. The mean and standard deviation of AUCs showed as the criterion for the 3-flod cross-validation training process, with the total number of training samples, responders (R) and non-responder (NR). (**c**) Pathway-based super signatures (PASS-PRE) consists of six selected pathways associate with the effect sizes (variable weights) from ENLR model. **(d)** Enrichment plot of pathways, (1) Complement cascade; (2) Regulation of insulin like growth factor IGF transport and uptake by insulin like growth factor binding proteins IGFBPS; (3) Binding and uptake of ligands by scavenger receptors; (4) Plasma lipoprotein remodeling; (5) Interleukin 2 family signaling; and (6) RA biosynthesis pathways. (**e**) ROC and AUC of PASS-PRE on pre-treatment samples from Van Allen et al. and Hugo et al. cohorts. (**f-i**) The Kaplan–Meier analysis of progression-free survival of pre-treatment samples in the combined test pre-treatment samples (**f**), Gide et al. cohort (**g**), Lee et al. cohort (**h**) and MGH cohort (**i**). The two-sided log-rank test compared high and low subgroups based on the mean of pre-treatment samples odd ratio as cutoff. Hazard ratio (HR) was calculated and shown with confidence interval (CI). Source data are provided as a Source Data file.

Supplementary Figure 2

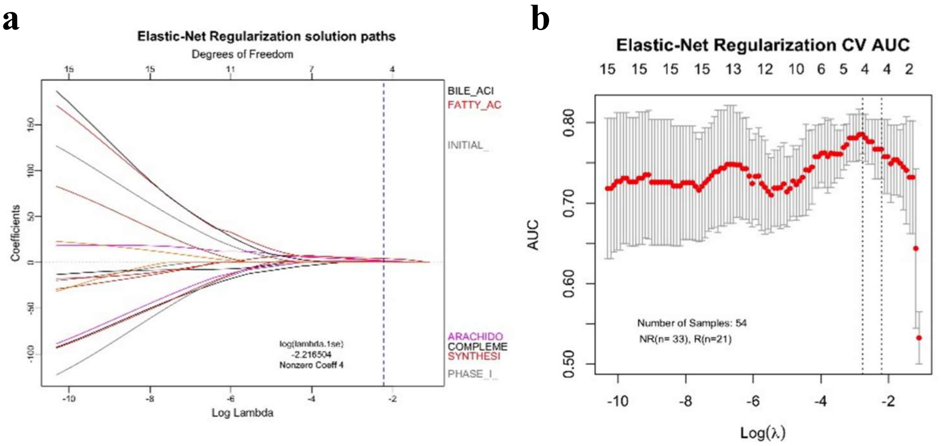

c

PASS-ON Model Effect Size Of Candidate Pathway

Pathway	Weight
Intercept	-1.32289
PEROXISOMAL LIPID METABOLISM	1.81734
GENERATION OF SECOND MESSENGER MOLECULES	1.92997
FATTY ACID METABOLISM	4.30198
PD 1 SIGNALING	1.15131

Only shows pathways which effect size is not zero

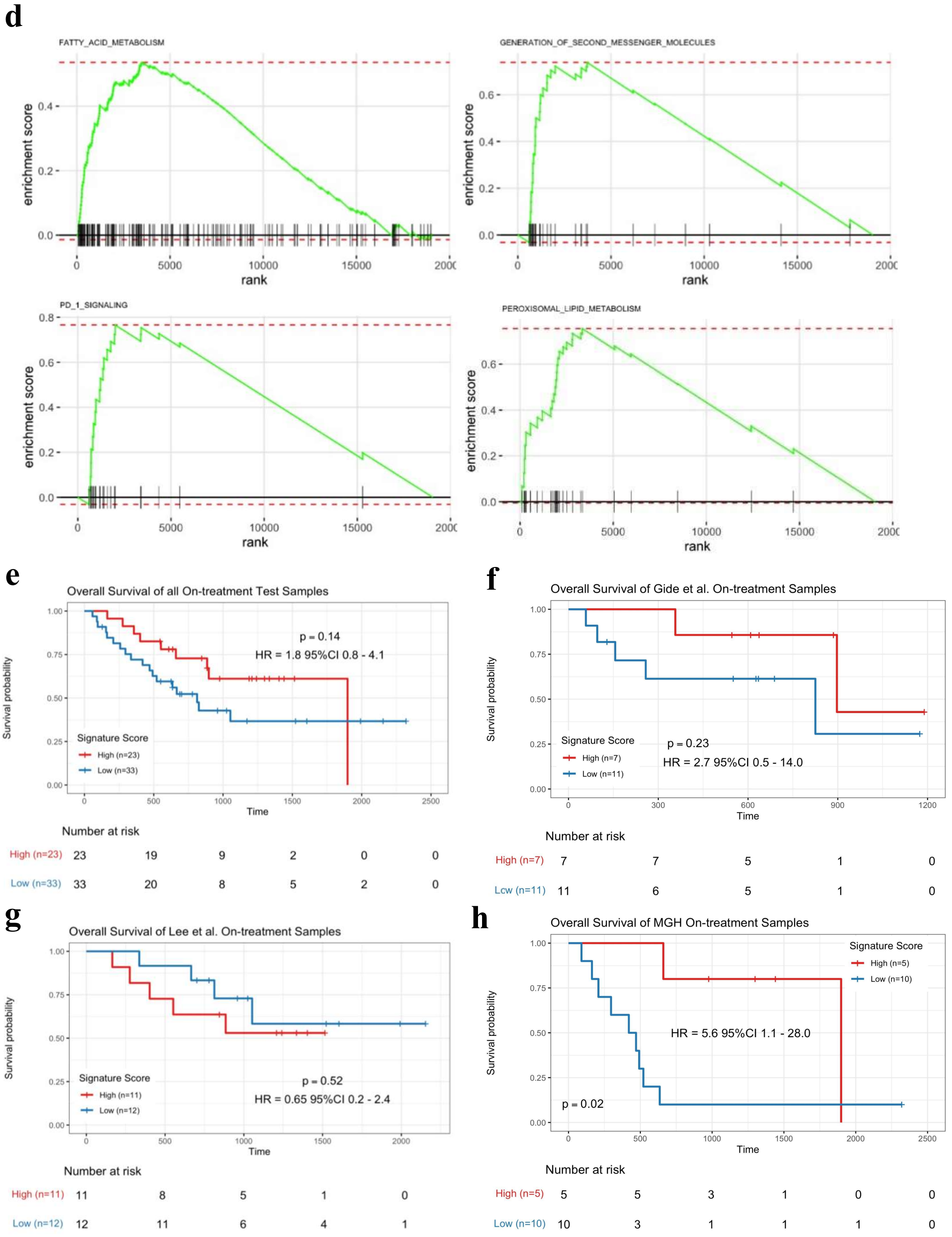

Supplementary Figure 2. Pathway-based Super Signature for On-treatment Samples. (**a, b**) The ENLR model's training parameter selection process on Riaz et al. on-treatment samples to generate PASS-ON signature, and each candidate pathways selection path associate with coefficient. The mean and standard deviation of AUCs showed as the criterion for the 3-flod cross-validation training process, with the total number of training samples, responders (R) and non-responder (NR). (**c**) Pathway-based super signatures (PASS-ON) consists of four selected pathways associate with the effect sizes (variable weights) from ENLR model. **(d)** Enrichment plot of pathways, (1) Peroxisomal Lipid Metabolism; (2) Generation of Second Messenger Molecules; (3) Fatty Acid Metabolism; and (4) PD1 Signaling (**e-h**) The Kaplan–Meier analysis of progression-free survival of on-treatment samples in the combined test on-treatment samples (**e**), Gide et al. cohort (**f**), Lee et al. cohort (**g**) and MGH cohort (**h**). Each Kaplan–Meier survival analysis using mean value of samples' odd ratio as cutoff to separate pre-treatment samples into high and low groups. The two-sided log-rank test compared high and low subgroups. Hazard ratio (HR) was calculated and shown with confidence interval (CI). Source data are provided as a Source Data file.

Supplementary Figure 3

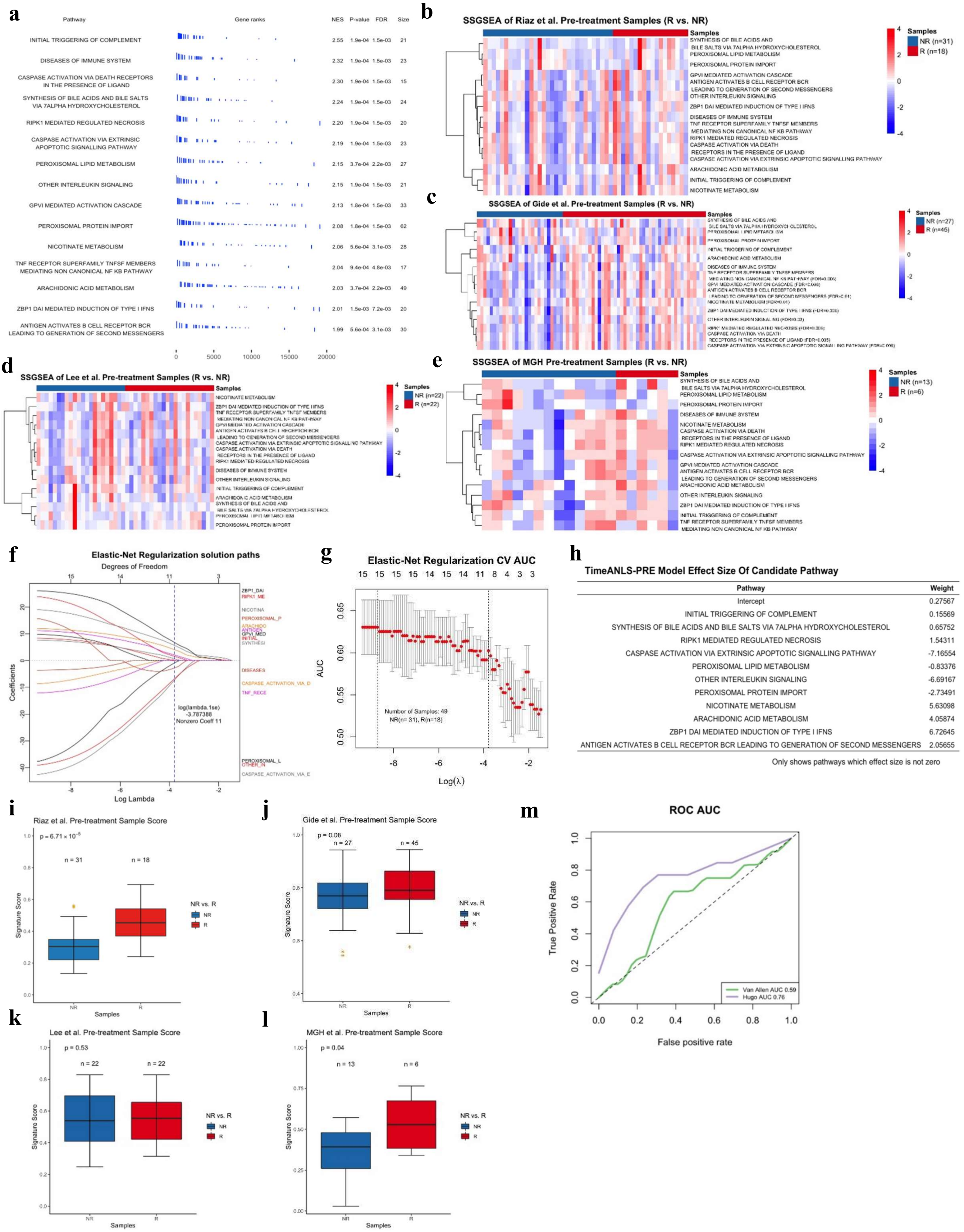

TimeANLS-PRE Model Effect Size Of Candidate Pathway

Pathway	Weight
Intercept	0.27567
INITIAL TRIGGERING OF COMPLEMENT	0.15569
SYNTHESIS OF BILE ACIDS AND BILE SALTS VIA 7ALPHA HYDROXYCHOLESTEROL	0.65752
RIPK1 MEDIATED REGULATED NECROSIS	1.54311
CASPASE ACTIVATION VIA EXTRINSIC APOPTOTIC SIGNALLING PATHWAY	-7.16554
PEROXISOMAL LIPID METABOLISM	-0.83376
OTHER INTERLEUKIN SIGNALING	-6.69167
PEROXISOMAL PROTEIN IMPORT	-2.73491
NICOTINATE METABOLISM	5.63098
ARACHIDONIC ACID METABOLISM	4.05874
ZBP1 DAI MEDIATED INDUCTION OF TYPE I IFNS	6.72645
ANTIGEN ACTIVATES B CELL RECEPTOR BCR LEADING TO GENERATION OF SECOND MESSENGERS	2.05655

Only shows pathways which effect size is not zero

Supplementary Figure 3. Time-response Interaction Pathway-based Super Signatures for Pre-treatment Samples. (**a**) GSEA results of 15 candidate pathways. Normalized Enrichment Score (NES) and the size of leading-edge gene set are calculated. The permutation based P-value shows the statistical significance of the enrichment score. The number of permutation is 10,000. False discovery rate (FDR) is the estimated probability that the normalized enrichment score represents a false positive finding. (**b-e**) The heatmap of ssGSEA values of responders (R) and non-responder(NR) pre-treatment samples in the Riaz et al. cohort (**b**), Gide et al. cohort (**c**), Lee et al. cohort (**d**). and MGH cohort (**e**). Non-responders are presented on the left side, and responders are presented on the right side. FDR-corrected two-sided Welch t-test was conducted to compare ssGSEA values between R and NR samples, only FDR < 0.05 showed here. **(f, g)** The ENLR model's training parameter selection process on Riaz et al. pre-treatment samples to generate TimeANLS-PRE signature, and each candidate pathways selection path associate with coefficient. The mean and standard deviation of AUCs showed as the criterion for the 3-flod cross-validation training process, with the total number of training samples, responders (R) and non-responder (NR). (**h)** Pathway-based super signatures (TimeANLS-PRE) consists of eleven selected pathways associate with the effect sizes (variable weights) from ENLR model. (**i-l**) The boxplot of pre-treatment samples' TimeANLS-PRE signature scores of responders (R) and non-responders(NR) in the Riaz et al. (**i**), Gide et al. cohort (**j**), Lee et al. cohort (**k**). and MGH cohort (**l**). The pre-treatment samples were separated into responders (R) and non-responders (NR) with number of samples showed in each cohorts. The P values were computed via a one-sided rank-sum test. Boxplot center lines indicate medians, box edges represent the interquartile range, whiskers extend to the minimum and maximum, and the outliers are plotted individually using the '*' symbol. (**m**) ROC and AUC of TimeANLS-PRE on pre-treatment samples from Van Allen et al. and Hugo et al. cohorts. Source data are provided as a Source Data file.

Supplementary Figure 4

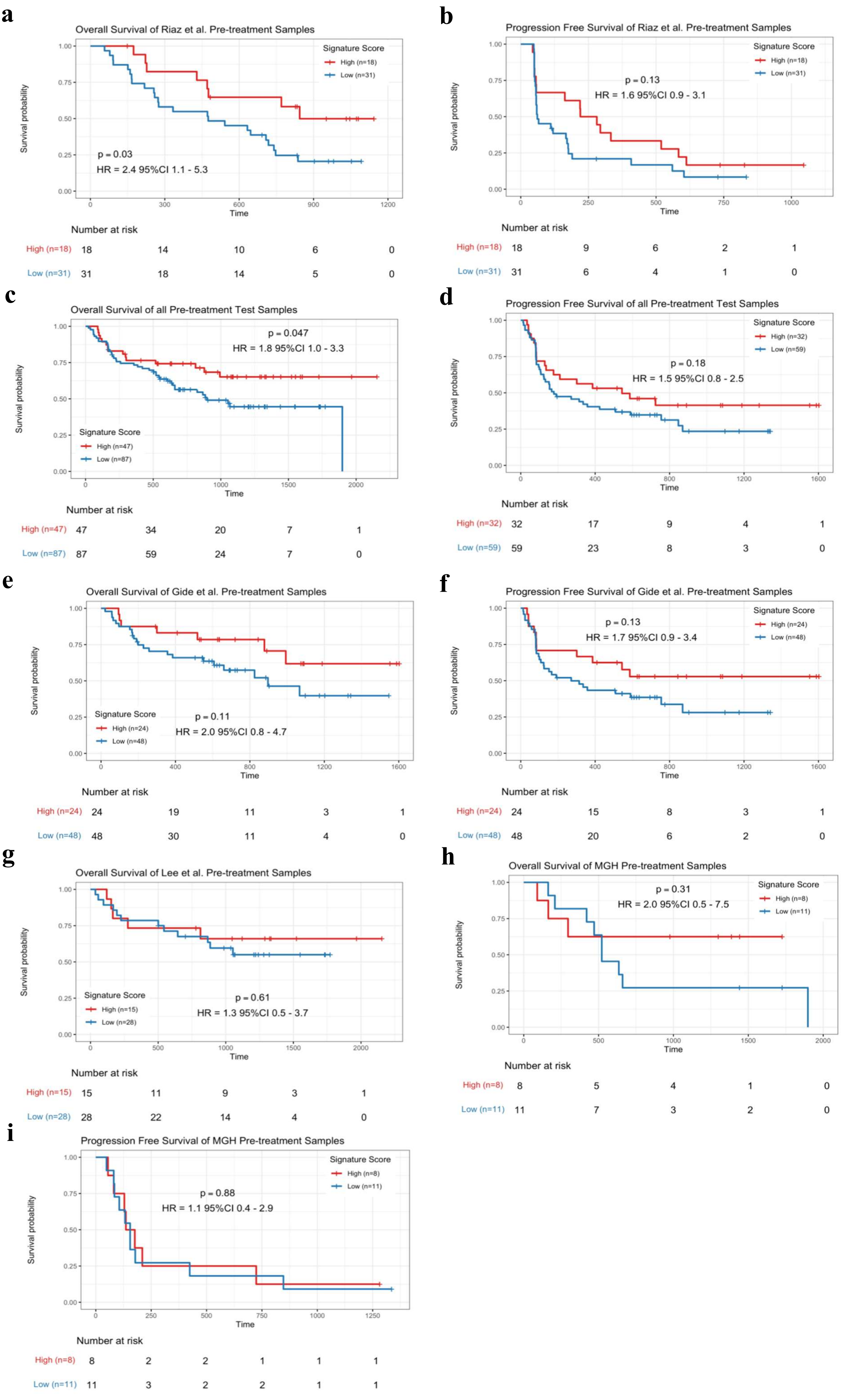

Supplementary Figure 4. Time-response Interaction Pathway-based Super Signatures for Pre-treatment Samples. (**a, b**) Overall survival analysis and progression free survival analysis of Riaz et al. pre-treatment samples (**c, d**) Overall survival analysis and progression free survival analysis of all test pre-treatment samples, which combined with Gide et al. cohort, Lee et al. cohort. and MGH cohort. (**e, f**) Overall survival analysis and progression free survival analysis of Gide et al. pre-treatment samples. (**g**) Overall survival analysis and progression free survival analysis of Lee et al. pre-treatment samples (**h, i**) Overall survival analysis and progression free survival analysis of MGH cohort. Each Kaplan–Meier survival analysis using mean value of samples' odd ratio as cutoff to separate pre-treatment samples into high and low groups. The two-sided log-rank test compared high and low subgroups. Hazard ratio (HR) was calculated and shown with confidence interval (CI). Source data are provided as a Source Data file.

Supplementary Figure 5

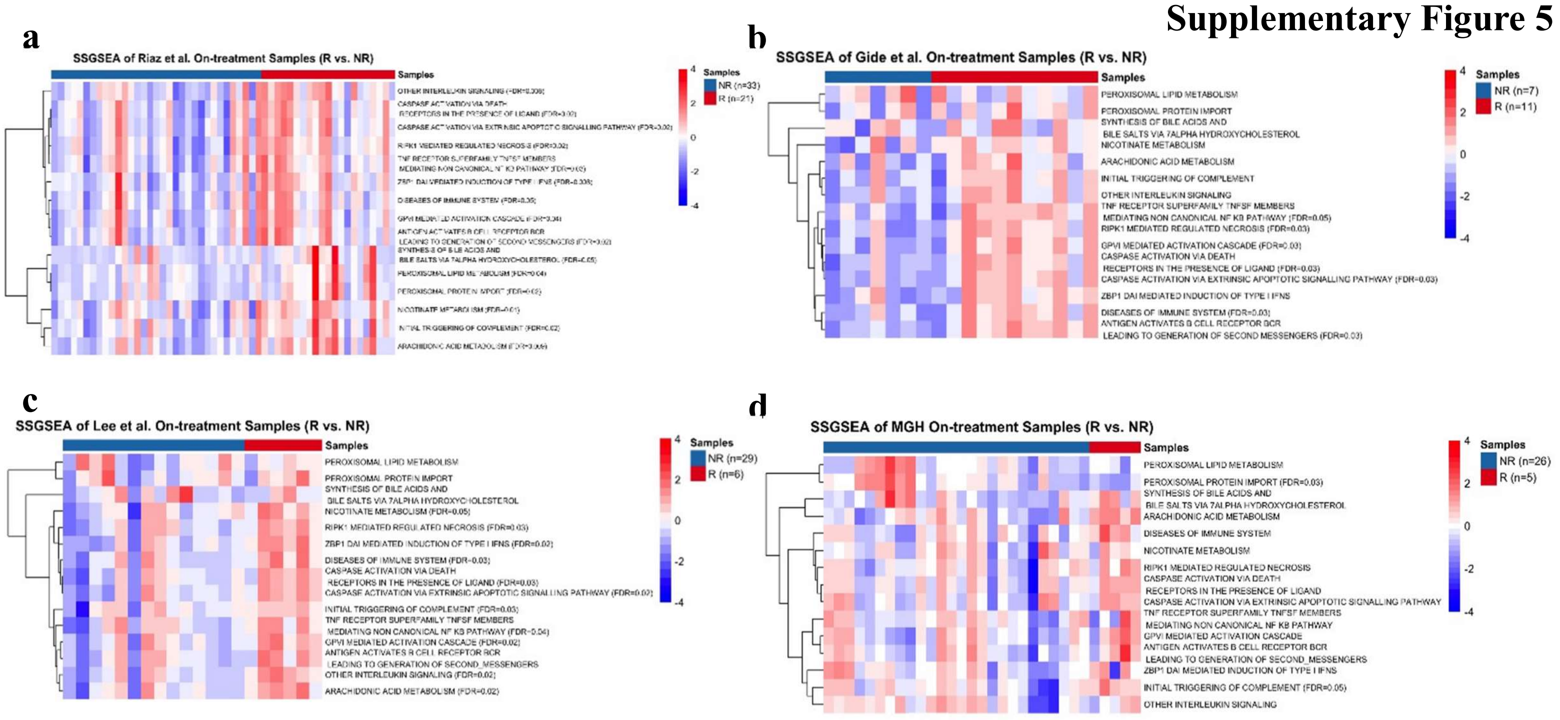

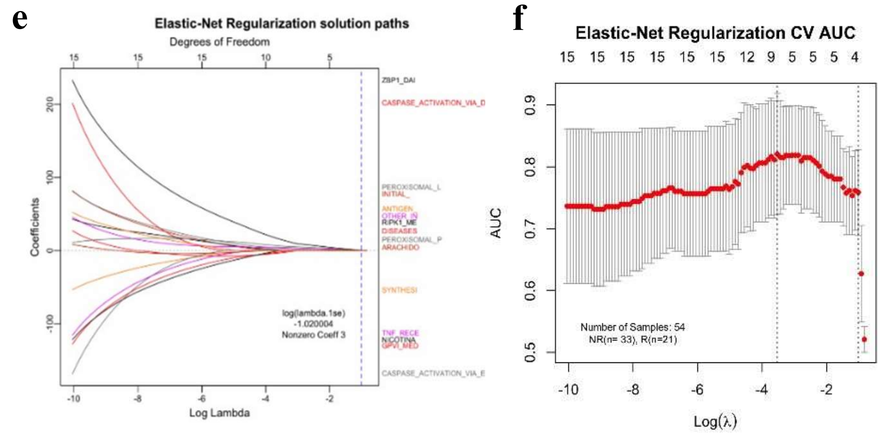

g

TimeANLS-ON Model Effect Size Of Candidate Pathway

Pathway	Weight
Intercept	0.27242
OTHER INTERLEUKIN SIGNALING	0.46901
ARACHIDONIC ACID METABOLISM	0.43386
ZBP1 DAI MEDIATED INDUCTION OF TYPE I IFNS	0.22165

Only shows pathways which effect size is not zero

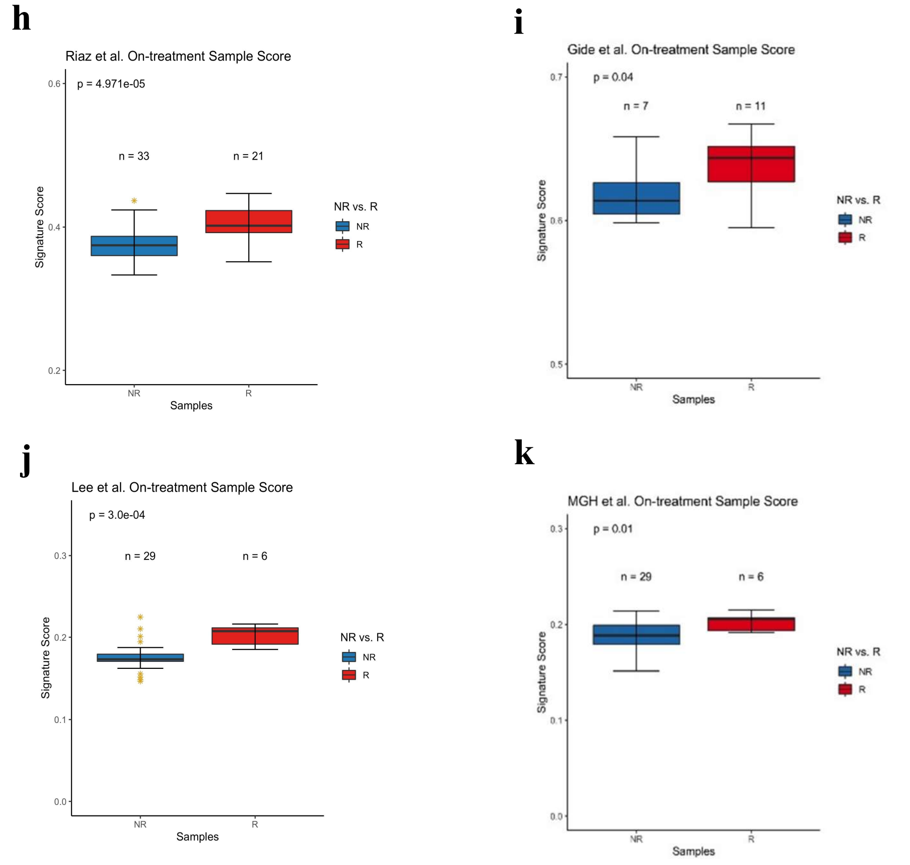

Supplementary Figure 5. Time-response Interaction Pathway-based Super Signatures for On-treatment (**a-d**) The heatmap of ssGSEA values of responders (R) and non-responder(NR) pre-treatment samples in the Riaz et al. cohort (**a**), Gide et al. cohort (**b**), Lee et al. cohort (**c**). and MGH cohort (**d**). Non-responders are presented on the left side, and responders are presented on the right side. FDR-corrected two-sided Welch t-test was conducted to compare ssGSEA values between R and NR samples, only FDR < 0.05 showed here.**(e, f)** The ENLR model's training parameter selection process on Riaz et al. on-treatment samples to generate TimeANLS-ON signature, and each candidate pathways selection path associate with coefficient. The mean and standard deviation of AUCs showed as the criterion for the 3-flod cross-validation training process, with the total number of training samples, responders (R) and non-responder (NR). (**g)** Pathway-based super signatures (TimeANLS-ON) consists of three selected pathways associate with the effect sizes (variable weights) from ENLR model. (**h-k**) The boxplot of on-treatment samples' TimeANLS-ON signature scores in the Riaz et al. (**H**), Gide et al. cohort (**i**), Lee et al. cohort (**j**). and MGH cohort (**k**). The pre-treatment samples were separated into responders (R) and non-responder (NR) with number of samples showed in each cohort. The P value was computed via a one-sided rank-sum test. Boxplot center lines indicate medians, box edges represent the interquartile range, whiskers extend to the minimum and maximum, and the outliers are plotted individually using the '*' symbol. (**f**) ROC and AUC of PASS-ON on the on-treatment samples in the Riaz et al. cohort. Source data are provided as a Source Data file.

Supplementary Figure 6

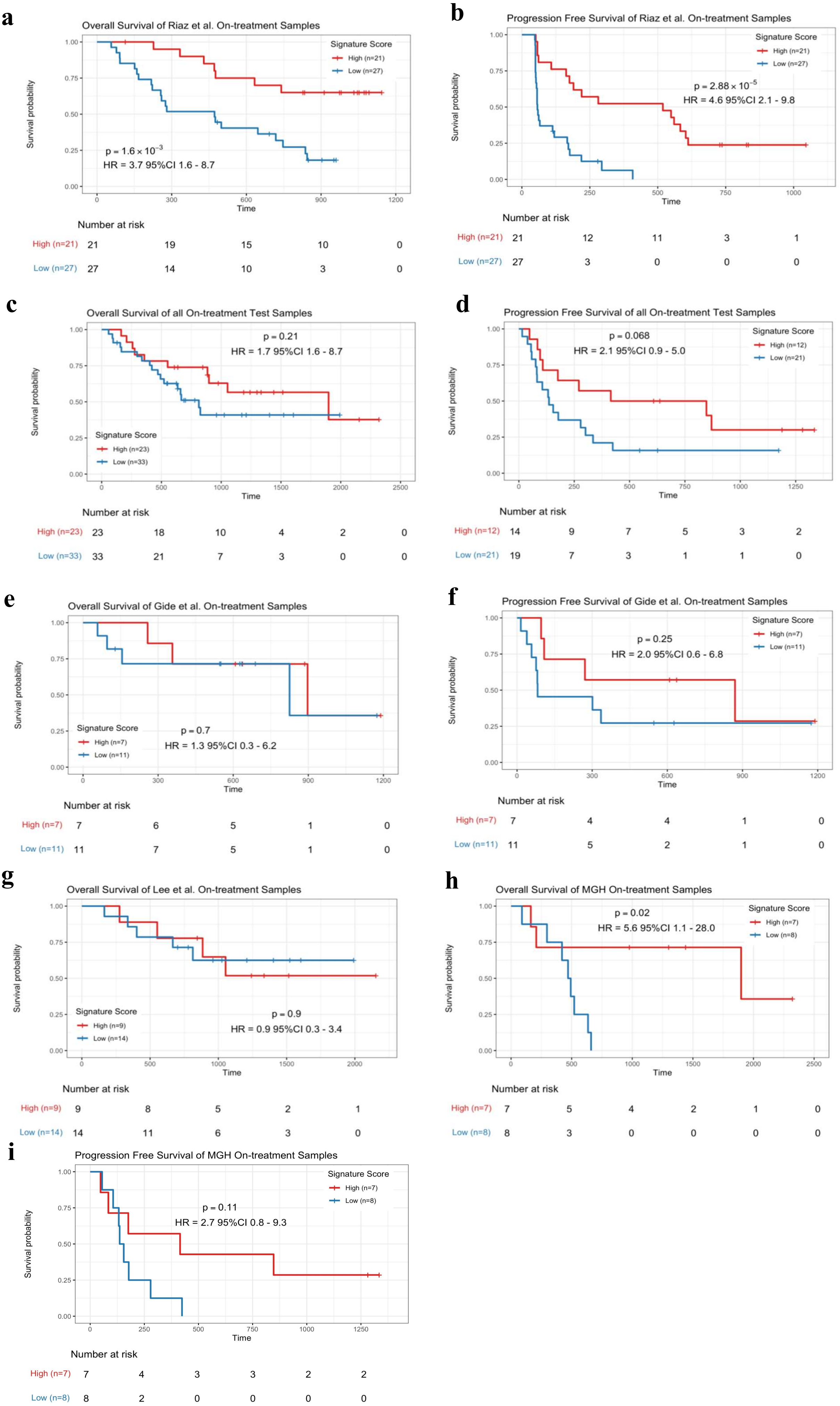

Supplementary Figure 6. Time-response Interaction Pathway-based Super Signatures for On-treatment Samples. (**a, b**) Overall survival analysis and progression free survival analysis of Riaz et al. on-treatment samples (**c, d**) Overall survival analysis and progression free survival analysis of all test on-treatment samples, which combined with Gide et al. cohort, Lee et al. cohort. and MGH cohort. (**e, f**) Overall survival analysis and progression free survival analysis of Gide et al. on-treatment samples. (**g**) Overall survival analysis and progression free survival analysis of Lee et al. on-treatment samples (**h, i**) Overall survival analysis and progression free survival analysis of MGH cohort. Each Kaplan–Meier survival analysis using mean value of samples' odd ratio as cutoff to separate pre-treatment samples into high and low groups. The two-sided log-rank test compared high and low subgroups. Hazard ratio (HR) was calculated and shown with confidence interval (CI). Source data are provided as a Source Data file.

a

Comparing the performance of each predictor with PASS-PRE model

Empirical P value (n=1000)

Signatures	Riaz	Gide	Lee	MGH	Mean.AUC
IFN_γ_Signature	0.000000e+00	1.0000000	1.000000e+00	9.475288e-119	6.409868e-65
T_cell_inflamed_GEP	7.597725e-300	1.0000000	9.999930e-01	1.775994e-19	7.708563e-25
Chemokine_Signature	0.000000e+00	1.0000000	1.000000e+00	1.185967e-20	2.396806e-62
Immunoscore	0.000000e+00	1.0000000	1.000000e+00	2.365716e-122	7.666701e-190
CYT	0.000000e+00	1.0000000	1.000000e+00	3.890527e-124	7.746975e-105
MHC_I	0.000000e+00	1.0000000	1.000000e+00	4.134693e-01	6.461049e-24
MHC_II	0.000000e+00	0.9946015	1.071298e-19	6.618574e-03	2.667031e-201
CD8Ratio	0.000000e+00	1.0000000	1.000000e+00	6.107136e-90	1.301723e-11
CD8+ T Cells Cibersort	0.000000e+00	1.0000000	1.000000e+00	3.472380e-136	1.074957e-174
IMPRES	0.000000e+00	0.0000000	4.636999e-01	4.731269e-144	0.000000e+00

b

Comparing the performance of each predictor with PASS-ON model

Empirical P value (n=1000)

Signatures	Riaz	Gide	Lee	MGH	Mean.AUC
IFN_γ_Signature	0	1.000000e+00	5.763803e-76	3.760601e-252	0.000000e+00
T_cell_inflamed_GEP	0	1.000000e+00	1.456067e-01	1.212660e-187	8.335913e-282
Chemokine_Signature	0	2.735093e-10	1.000000e+00	1.689015e-283	0.000000e+00
Immunoscore	0	1.000000e+00	1.124501e-50	0.000000e+00	0.000000e+00
CYT	0	5.587871e-01	1.368044e-194	1.918949e-218	0.000000e+00
MHC_I	0	1.000000e+00	0.000000e+00	0.000000e+00	0.000000e+00
MHC_II	0	9.233660e-27	1.506653e-74	0.000000e+00	0.000000e+00
CD8Ratio	0	1.000000e+00	0.000000e+00	0.000000e+00	0.000000e+00
CD8+ T Cells Cibersort	0	3.907311e-40	0.000000e+00	0.000000e+00	0.000000e+00
IMPRES	0	1.630198e-235	3.456735e-120	1.000000e+00	0.000000e+00

Supplementary Figure 7. Our signature compared with other published signature (a) one-sided rank sum test between PASS-PRE AUCs and other published signatures' AUCs. **(b)** one-sided rank sum test between PASS-ON AUCs and other published signatures' AUCs. The empirical P values comparing our signature performance to that of each of the other public existing signature and for the aggregate of all datasets (Each time randomly selected 80% responders and non-responders samples and calculate each signature AUC value. The process was repeated 1000 times. The P-value which less than 0.001 denotes that the prediction performance of our signature was superior to that of compared predictor).
