# Supplementary material for: Pathway signatures derived from on-treatment tumor specimens predict response to anti-PD1 blockade in metastatic melanoma
Source: Nat Commun. 2021 Oct 15;12:6023. doi: 10.1038/s41467-021-26299-4 (PMC8519947; doi:10.1038/s41467-021-26299-4)
Supplement: Supplementary file 2 — Description of Additional Supplementary Files [file 41467_2021_26299_MOESM2_ESM.docx]

**Description of Additional Supplementary Files**

**Supplementary Data 1.**

Clinical information summary of patients in the Riaz et al. (a), Gide et al. (b), Lee et al. (c) and MGH (d) datasets.

**Supplementary Data 2.**

Differential gene expression between responder and non-responder at pre-treatment time point in Riaz et al. (a), Gide et al. (b), Lee et al. (c) and MGH (d) datasets.

**Supplementary Data 3.**

ssGSEA pathway scores based on responder vs non-responder analysis and PASS-PRE signature scores in pre-treatment samples in Gide et al. (a, b), Lee et al. (c, d) and MGH (e, f) datasets.

**Supplementary Data 4.**

Differential gene expression (a), gene set enrichment analysis (b), ssGSEA pathway scores (c) and PASS-ON signature scores (d) between responder and non-responder at on-treatment time point in Riaz et al. dataset.

**Supplementary Data 5.**

ssGSEA pathway scores and PASS-ON signature scores based on responder vs non-responder analysis in on-treatment samples in Gide et al. (a, b), Lee et al. (c, d) and MGH (e, f) datasets.

**Supplementary Data 6.**

Differential gene expression (a) and gene set enrichment analysis (b) between pre- and on-treatment biopsy samples of responders in Riaz et al. dataset.

**Supplementary Data 7.**

ssGSEA pathway scores based on pre- vs on-treatment analysis in pre-treatment samples in Riaz et al. (a), Gide et al. (b), Lee et al. (c) and MGH (d) datasets.

**Supplementary Data 8.**

Time-response interaction signature scores of pre-treatment samples in Riaz et al. (a), Gide et al. (b), Lee et al. (c) and MGH (d) datasets.

**Supplementary Data 9.**

ssGSEA pathway scores based on pre- vs on-treatment analysis in on-treatment samples in Riaz et al. (a), Gide et al. (b), Lee et al. (c) and MGH (d) datasets.

**Supplementary Data 10.**

Time-response interaction signature scores of on-treatment samples in Riaz et al. (a), Gide et al. (b), Lee et al. (c) and MGH (d) datasets.
