# Supplementary material for: Pathway signatures derived from on-treatment tumor specimens predict response to anti-PD1 blockade in metastatic melanoma
Source: Nat Commun. 2021 Oct 15;12:6023. doi: 10.1038/s41467-021-26299-4 (PMC8519947; doi:10.1038/s41467-021-26299-4)
Supplement: Supplementary file 13 — Reporting Summary [file 41467_2021_26299_MOESM13_ESM.pdf]

nature portfolio

Corresponding author(s): Gao Zhang
Last updated by author(s): Aug 24, 2021

Reporting Summary

Nature Portfolio wishes to improve the reproducibility of the work that we publish. This form provides structure for consistency and transparency in reporting. For further information on Nature Portfolio policies, see our Editorial Policies and the Editorial Policy Checklist.

Statistics

For all statistical analyses, confirm that the following items are present in the figure legend, table legend, main text, or Methods section.

n/a	Confirmed	
☐	☒	The exact sample size (*n*) for each experimental group/condition, given as a discrete number and unit of measurement
☐	☒	A statement on whether measurements were taken from distinct samples or whether the same sample was measured repeatedly
☐	☒	The statistical test(s) used AND whether they are one- or two-sided<br>*Only common tests should be described solely by name; describe more complex techniques in the Methods section.*
☒	☐	A description of all covariates tested
☒	☐	A description of any assumptions or corrections, such as tests of normality and adjustment for multiple comparisons
☐	☒	A full description of the statistical parameters including central tendency (e.g. means) or other basic estimates (e.g. regression coefficient) AND variation (e.g. standard deviation) or associated estimates of uncertainty (e.g. confidence intervals)
☐	☒	For null hypothesis testing, the test statistic (e.g. *F*, *t*, *r*) with confidence intervals, effect sizes, degrees of freedom and *P* value noted<br>*Give P values as exact values whenever suitable.*
☒	☐	For Bayesian analysis, information on the choice of priors and Markov chain Monte Carlo settings
☒	☐	For hierarchical and complex designs, identification of the appropriate level for tests and full reporting of outcomes
☒	☐	Estimates of effect sizes (e.g. Cohen's *d*, Pearson's *r*), indicating how they were calculated

Our web collection on statistics for biologists contains articles on many of the points above.

Software and code

Policy information about availability of computer code

Data collection	Illumina HiSeq2000 and Illumina NextSeq 500
Data analysis	Differential expression analysis used DESeq2, an R-package version 1.30.1. Gene set enrichment analysis (GSEA) was implemented by using fgsea, an R-package version 1.16.0. The R-package, GSVA (version 1.38.2) computed the single sample GSEA (ssGSEA) value of each selected pathways. Elastic-Net penalized logistic regression model implemented by R-package glmnet (version 4.1-1), and was used to determine the most prognostic signatures from the candidate pathways. FDR-corrected two-sided Welch t-test was conducted to compare ssGSEA values between R and NR samples. One-sided rank sum test was conducted to compare the signature scores between R and NR. Survival analysis was conducted with the Kaplan–Meier method, and samples were separated into high and low group use mean value of samples' odd ratio as cutoff. The log-rank test was used to determine statistical significance. Hazard ratio (HR) was calculated using cox regression, while we found that the proportional Hazards assumption generally held well in most datasets. Survival analysis implement in R-packages, survival(version 3.2-7) and survminer(version 0.4.9). Code is public available on Github:https://github.com/dukekuang/PASS-ON-codes.

For manuscripts utilizing custom algorithms or software that are central to the research but not yet described in published literature, software must be made available to editors and reviewers. We strongly encourage code deposition in a community repository (e.g. GitHub). See the Nature Portfolio guidelines for submitting code & software for further information.

Data

Policy information about availability of data

All manuscripts must include a data availability statement. This statement should provide the following information, where applicable:

- Accession codes, unique identifiers, or web links for publicly available datasets
- A description of any restrictions on data availability
- For clinical datasets or third party data, please ensure that the statement adheres to our policy

The transcriptomic data of the newly MGH patients generated in this study have been deposited in the GEO database under accession code GSE168204 [https://www.ncbi.nlm.nih.gov/geo/query/acc.cgi?acc=GSE168204]. All patients' data analyzed from published papers are referenced to and publicly available accordingly. The Riaz et al. data used in this study are available in the GEO database under accession code GSE91061 [https://www.ncbi.nlm.nih.gov/geo/query/acc.cgi?acc=GSE91061]. The Gide et al. data used in this study are available in the BioProject database under accession code PRJEB23709 [https://www.ebi.ac.uk/ena/browser/view/PRJEB23709]. The Lee et al. data used in this study are available in the EGA database under accession code EGAD00001005738 [https://ega-archive.org/datasets/EGAD00001005738]. The published MGH data used in this study are available in the GEO database under accession code GSE115821 [https://www.ncbi.nlm.nih.gov/geo/query/acc.cgi?acc=GSE115821]. The Hugo et al. data used in this study are available in the GSE database under accession code GSE78220 [https://www.ncbi.nlm.nih.gov/geo/query/acc.cgi?acc=GSE78220]. The Van Allen et al. data are available under restricted access, access can be obtained by dbGaP study accession phs000452.v2.p1 [https://www.ncbi.nlm.nih.gov/projects/gap/cgi-bin/study.cgi?study_id=phs000452.v2.p1].

Field-specific reporting

Please select the one below that is the best fit for your research. If you are not sure, read the appropriate sections before making your selection.

[x] Life sciences [ ] Behavioural & social sciences [ ] Ecological, evolutionary & environmental sciences

For a reference copy of the document with all sections, see nature.com/documents/nr-reporting-summary-flat.pdf

Life sciences study design

All studies must disclose on these points even when the disclosure is negative.

Sample size	Only pre- and on-treatment samples that were available for this study were included in training and testing analysis. No specific sample size calculation was performed, and we included eligible samples as many as possible in this study.
Data exclusions	In this study, those patients' tumor specimens which were not subject to RNA sequencing, lacked response evaluation or those patients with duplicated tumor specimens of the same time point were excluded for further analysis.
Replication	We tested and evaluated the models using additional independent datasets to replicate the prediction performance.
Randomization	We randomly splited samples into different sets for training, validation and testing.
Blinding	The response labels were hold blind to the machine learning models under evaluation.

Reporting for specific materials, systems and methods

We require information from authors about some types of materials, experimental systems and methods used in many studies. Here, indicate whether each material, system or method listed is relevant to your study. If you are not sure if a list item applies to your research, read the appropriate section before selecting a response.

Materials & experimental systems

n/a	Involved in the study	
[x]	[ ]	Antibodies
[x]	[ ]	Eukaryotic cell lines
[x]	[ ]	Palaeontology and archaeology
[x]	[ ]	Animals and other organisms
[ ]	[x]	Human research participants
[x]	[ ]	Clinical data
[x]	[ ]	Dual use research of concern

Methods

n/a	Involved in the study	
[x]	[ ]	ChIP-seq
[x]	[ ]	Flow cytometry
[x]	[ ]	MRI-based neuroimaging

Human research participants

Policy information about studies involving human research participants

Population characteristics	In the newly generated cohort, 19 tumor biopsies were derived from metastatic melanoma patients treated with anti-PD1/PD-L1 and 8 tumor specimens were obtained from metastatic melanoma patients treated with anti-PD1+anti-CTLA4. The age of included patients ranged from 21 to 81 years old (average age of 62 years old). 6 (28.6%) samples were obtained from female patients and 21 (71.4%) samples were obtained from male patients.
Recruitment	The samples were compiled retrospectively. No active recruitment were conducted during this study.
Ethics oversight	In the newly generated cohort, patient samples were collected under the Institutional Review Board (IRB) protocols of Dana-Farber Cancer Institute (protocol 11-181) and The Wistar Institute (Human subjects protocol 2802240).

Note that full information on the approval of the study protocol must also be provided in the manuscript.
